# Peripheral immune profiling of soft tissue sarcoma: perspectives for disease monitoring

**DOI:** 10.3389/fimmu.2024.1391840

**Published:** 2024-10-21

**Authors:** Jani Sofia Almeida, Luana Madalena Sousa, Patrícia Couceiro, Tânia Fortes Andrade, Vera Alves, António Martinho, Joana Rodrigues, Ruben Fonseca, Paulo Freitas-Tavares, Manuel Santos-Rosa, José Manuel Casanova, Paulo Rodrigues-Santos

**Affiliations:** ^1^ Center for Neurosciences and Cell Biology (CNC), Laboratory of Immunology and Oncology, University of Coimbra, Coimbra, Portugal; ^2^ Faculty of Medicine (FMUC), Institute of Immunology, University of Coimbra, Coimbra, Portugal; ^3^ Center for Investigation in Environment, Genetics and Oncobiology (CIMAGO), University of Coimbra, Coimbra, Portugal; ^4^ Coimbra Institute for Clinical and Biomedical Research (iCBR), University of Coimbra, Coimbra, Portugal; ^5^ Center for Innovation in Biomedicine and Biotechnology (CIBB), University of Coimbra, Coimbra, Portugal; ^6^ Clinical and Academic Centre of Coimbra (CACC), Coimbra, Portugal; ^7^ Portuguese Institute for Blood and Transplantation (IPST), Blood and Transplantation Center of Coimbra, Coimbra, Portugal; ^8^ Tumor Unit of the Locomotor Apparatus, University Clinic of Orthopedics, Orthopedics Oncology Service, Coimbra Hospital and Universitary Centre (CHUC), Coimbra, Portugal

**Keywords:** soft tissue sarcoma, immunophenotyping, gene expression profiling, cytokines, chemokines, growth factors, immune checkpoints

## Abstract

Studying the tumor microenvironment and surrounding lymph nodes is the main focus of current immunological research on soft tissue sarcomas (STS). However, due to the restricted opportunity to examine tumor samples, alternative approaches are required to evaluate immune responses in non-surgical patients. Therefore, the purpose of this study was to evaluate the peripheral immune profile of STS patients, characterize patients accordingly and explore the impact of peripheral immunotypes on patient survival. Blood samples were collected from 55 STS patients and age-matched healthy donors (HD) controls. Deep immunophenotyping and gene expression analysis of whole blood was analyzed using multiparametric flow cytometry and real-time RT-qPCR, respectively. Using xMAP technology, proteomic analysis was also carried out on plasma samples. Unsupervised clustering analysis was used to classify patients based on their immune profiles to further analyze the impact of peripheral immunotypes on patient survival. Significant differences were found between STS patients and HD controls. It was found a contraction of B cells and CD4 T cells compartment, along with decreased expression levels of ICOSLG and CD40LG; a major contribution of suppressor factors, as increased frequency of M-MDSC and memory Tregs, increased expression levels of ARG1, and increased plasma levels of IL-10, soluble VISTA and soluble TIMD-4; and a compromised cytotoxic potential associated with NK and CD8 T cells, namely decreased frequency of CD56^dim^ NK cells, and decreased levels of PRF1, GZMB, and KLRK1. In addition, the patients were classified into three peripheral immunotype groups: "immune-high," "immune-intermediate," and "immune-low." Furthermore, it was found a correlation between these immunotypes and patient survival. Patients classified as "immune-high" exhibited higher levels of immune-related factors linked to cytotoxic/effector activity and longer survival times, whereas patients classified as "immune-low" displayed higher levels of immune factors associated with immunosuppression and shorter survival times. In conclusion, it can be suggested that STS patients have a compromised systemic immunity, and the correlation between immunotypes and survival emphasizes the importance of studying peripheral blood samples in STS. Assessing the peripheral immune response holds promise as a useful method for monitoring and forecasting outcomes in STS.

## 1 Introduction

Soft tissue sarcomas (STS) represent a broad class of rare and highly heterogeneous mesenchymal tumors. The estimated incidence is 1.5–3.0 times per 100,000 individuals annually, and the World Health Organization (WHO) documented over 100 histopathological subtypes in 2020 (1–3). The main concerns in STS, given the 20% 5-year survival rate for advanced cancer, is the rate of recurrence and metastatic disease, which presents a treatment challenge (3–5). Consequently, it is essential to regularly monitor STS patients in order to forecast the course of the disease. To follow up with STS patients, clinical practice currently uses imaging methods and evaluates general cancer biomarkers (6, 7). But since there aren’t any particular biomarkers for STS used in clinical care, there’s an opportunity to look into and find cellular and molecular factors that can be used to aid doctors in clinical management.

Research on immune-related parameters as possible indicators of cancer development has increased dramatically as a result of immunotherapy’s advancements (8–10). This push for improved prognostic, diagnostic, and monitoring approaches in cancer has reignited interest in immunologic markers within STS. Inspired by William B. Coley’s early 20th-century work on immunotherapy in sarcomas (11), there is a growing recognition of the immune system’s critical role in STS. While STS has traditionally been viewed as “cold” tumors with limited immune response (12–14), emerging evidence challenges this perception. Recent studies have highlighted the variability in tumor mutational burden, the presence of an occasionally “hot” tumor microenvironment, and observed responses to immunotherapy, underscoring the complex and nuanced role of immunity in STS (15, 16).

The immune contexture in STS tumors is marked by specific features, including tumor-associated macrophages (TAM), dysfunctional tumor-infiltrating lymphocytes (TIL), reduced CD8 T cell and NK cell activity, increased Treg cells, limited B cell infiltration, and impaired dendritic cell (DC) function (17–20). Moreover, gene expression analysis of tumor samples in STS shown that a 20-gene signature related to cytotoxic immune response further strengthened the prognostic potential of the 67-gene Complexity Index in SARComas (CINSARC) transcriptomic signature, that is a promising predictor of metastatic disease in STS (21–23). Additionally, blood plasma cytokine analysis in STS has shown correlations with clinical parameters (24, 25), suggesting that plasma proteins could be valuable for patient stratification and monitoring. Recent studies also highlight the importance of tumor immunotypes, such as Sarcoma Immune Classes (SIC A-E), in predicting patient outcomes and potential responses to immunotherapy (18, 26, 27). Although understanding the tumor’s local immune status is crucial, challenges related to scheduling and sample availability can limit the effectiveness of monitoring systems that depend on tumor samples. Therefore, it is essential to explore alternative collection methods, such as analyzing peripheral blood samples.

Systemic immunity plays a critical role in cancer control, with changes in peripheral immune compositions both impacting and reflecting tumor responses (28). Alterations in circulating immune cells have been associated with prognosis across various cancer types, indicating their potential as survival markers (29–32). Although research on STS is limited, existing studies have shown that circulating immune cells, immune-related gene expression, and plasma cytokine levels hold promise for patient stratification (8, 23, 24, 33). These insights emphasize the need for comprehensive assessments of both cellular and molecular parameters in peripheral blood, which could help identify distinct immunotypes and provide a more nuanced understanding of a patient’s immune status. Consequently, investigating circulating biomarkers as predictors of disease outcomes in STS represents a valuable and promising area of research.

Hence, this study hypothesizes that peripheral immune profiles can function as biomarkers for distinguishing disease status and monitoring treatment responses in patients with STS. The research aims to evaluate the systemic immune compartment of STS patients, and assess the impact of histological classification, treatment response, and therapy. By analyzing immune cells, immune-related genes (IRG), and immune-related soluble factors (IRSF), the study seeks to identify distinct peripheral immunotypes associated with those variables. Additionally, the study explores the correlation of immunotype classification with survival outcome. It is anticipated that compromised systemic immunity in STS patients will be reflected in distinct immune profiles, which may vary in their impact on survival. The focus on systemic immune-related biomarkers represents a significant shift, introducing non-invasive, real-time monitoring methods that could transform the clinical approach to STS.

## 2 Materials and methods

### 2.1 STS patients and healthy donor controls

From November 2018 to February 2023, peripheral blood samples and clinical data were collected at the Tumor Unit of the Locomotor Apparatus, University Clinic of Orthopedics, Orthopedics Oncology Service, Coimbra Hospital and Universitary Centre, which is a European Reference Center for Adult STS Treatment. The inclusion criteria for patients were confirmed STS diagnostic, not including gastrointestinal tumor type (GIST), and age greater than 18 years. Patients with confirmed viral or bacterial infections were excluded from the study. A total of 55 STS patients’ peripheral blood samples and age-matched healthy donors (HD) controls were examined. For research involving human subjects, the World Medical Association’s Helsinki Declaration is adhered to in this work. All participants gave their informed consent after receiving thorough information regarding the goal of the study. The Coimbra Hospital and Universitary Center, Portugal, and the University of Coimbra’s Faculty of Medicine Ethics Committee provided ethical permission for this study, with references CE-018/2021 and CHUC-021-19, respectively. The study’s patient population’s clinical and demographic information was outlined in **Table 1** and in more detail in **Supplementary File S1**.

**Table 1:** Demographic and clinical characteristics of STS patients enrolled in the study.

Characteristic, unit	N (%) or mean (SD)
***Samples***	55 (100%)
Age, years	54 ± 15
Sex (% of females)	29 (52.7%)
Disease status	
Non-metastatic	20 (36.4%)
Primary	16 (29.1%)
Recurrence	4 (7.3%)
Metastatic	35 (63.6%)
Primary	25 (45.1%)
Recurrence	10 (18.2%)
Primary anatomical localization	
Extremity	22 (40.0%)
Upper limb	20 (36.4%)
Lower limb	2 (6.4%)
Trunk (not retroperitoneal)	16 (29.1%)
Thorax	5 (9.1%)
Pelvis	4 (7.3%)
Trunk, unspecified	3 (5.5%)
Heart	1 (1.8%)
Liver	1 (1.8%)
Jejunum	1 (1.8%)
Adrenal gland	1 (1.8%)
Retroperitoneal	7 (12.7%)
Gynecological region	9 (16.4%)
Uterus	8 (14.5%)
Spermatic cord	1 (1.8%)
Head and neck	1 (1.8%)
Lineage of cell differentiation	
Leiomyosarcoma	18 (32.7%)
Liposarcoma	9 (16.4%)
Undifferentiated sarcoma	9 (16.4%)
Synovial sarcoma	8 (14.5%)
Other	11 (20%)
Malignant peripheral nerve sheath tumor	3 (5.5%)
Haemangiosarcoma	2 (3.6%)
Clear cell sarcoma	2 (3.6%)
Alveolar soft part sarcoma	1 (1.8%)
Rhabdomyosarcoma	1 (1.8%)
Embryonal sarcoma	1 (1.8%)
Endometrial stromal sarcoma	1 (1.8%)
Treatment and response	
Surgery (diagnostic/recurrence)	8 (14.5%)
Stable disease (chemotherapy)	26 (47.3%)
Progression disease (chemotherapy)	21 (38.2%)
Therapy	
Anthracycline-based therapy	9 (16.4%)
Anthracycline-based therapy followed by trabectedin-based therapy	19 (34.5%)
Trabectedin-based therapy	8 (14.5%)
Other	11 (20%)
Not applicable	8 (14.5%)
Survival	
Alive with disease	31 (56.4%)
Dead of disease	20 (36.4%)
Dead of other causes	4 (7.3%)
Time after collection (TAC estimated, months)	15 ± 12 months
Time after diagnosis (TAD estimated, months)	42 ± 34 months

The patient cohort reflects the heterogeneous population encountered in real-world settings for STS. Therefore, patients were categorized by histological types, including leiomyosarcoma (LMS), liposarcoma (LS), undifferentiated sarcoma (US), synovial sarcoma (SS), and a miscellaneous “Other” group for histotypes with three or fewer patients. To analyze treatment impact and patient response, they were further divided into groups: those recommended for primary tumor surgery (DX), those with stable disease (SD), and those with disease progression (PD), with classifications made by the clinical team. Additionally, patients were sorted by their therapy regimen at the time of sample collection, resulting in four distinct groups: those receiving anthracycline-based therapy (ANTHRA), those on trabectedin-based therapy following anthracycline treatment (ANTHRA + TRAB), those on first-line trabectedin treatment (TRAB), and those following various other treatments (“OTHER”). Other factors such as gender, anatomical site, and presence of metastatic disease were also evaluated, but since no significant differences were found, these data are not included here (data not shown).

### 2.2 Peripheral immunophenotyping

For immunophenotyping, peripheral blood samples collected from 55 STS patients and 45 HD controls were analyzed. First, with the use of the hematological counter DxH500 (Beckman Coulter, Pasadena, CA, USA), the absolute frequency (AF) of total leucocytes (LEU) and the AF and relative frequency (RF) of the major LEU populations, such as lymphocytes (LY), monocytes (MO), and granulocytes (GR), were determined.

Then, peripheral blood samples were stained and prepared for flow cytometry analysis using an 8-color flow cytometer, BD FACSCanto II (BD Biosciences, San Jose, CA, USA), with BD FACSDiva software (BD Biosciences, San Jose, CA, USA). Initially, 100 µL of peripheral blood or up to 1x10^6^ LEU were incubated with fluorochrome-conjugated monoclonal antibodies (mAbs) for 15 minutes in the dark at room temperature. Following staining, red blood cells were lysed using 2 mL of BD Lysing Solution (BD Biosciences, San Jose, CA, USA) for 10 minutes under the same conditions. The samples were then centrifuged at 450 × *g* for 5 minutes; the supernatant was discarded, and the pellet was resuspended in 2 mL of 1x phosphate buffer saline (PBS) for washing. After a second centrifugation at 450 × *g* for 5 minutes, the supernatant was discarded, and the cells were resuspended in 1x PBS for acquisition.

The antibody panel employed, as previously described in the literature (34, 35), included 6 different combinations of fluorochrome-conjugated mAbs, enabling the identification of 83 immune cell populations. These included various lymphocyte subpopulations, dendritic cells (DC), and myeloid-derived suppressor cells (MDSC), along with key receptors related to cell maturation, activation, and suppression. The antibodies were titrated to determine the optimal concentration for up to 1x10^6^ LEU in 100 µL, with detailed antibody specifications provided in **Supplementary File S2**. Data analysis was performed using FlowJo v.10.7 software (BD Biosciences, Ashland, OR, USA), and the gating strategy is outlined in **Supplementary File S3**.

### 2.3 Whole blood immune-related gene expression profiling

Approximately 9 mL of whole blood from 55 STS patients and 45 HD controls was drawn into PAXgene Blood RNA Tubes^®^ (PreAnalytiX, Hombrechtikon, Switzerland), which stabilize and preserve RNA. After collection, the tubes were gently inverted to mix with the stabilization reagent and stored at room temperature for at least 2 hours. They were then frozen at -80°C until RNA extraction. RNA extraction was performed using the PAXgene Blood RNA Kit^®^ (PreAnalytiX, Hombrechtikon, Switzerland). The RNA PAXgene tubes were centrifuged at 3 000 × *g* for 10 minutes to pellet cellular components. The pellet was resuspended in RNase-free water, vortexed, and centrifuged again. The cell lysate was incubated with buffers and proteinase K at 55°C for 10 minutes, then homogenized using the PAXgene Shredder spin column. After adding absolute ethanol, the lysate was transferred to PAXgene RNA spin columns, where RNA was bound, washed, and treated with DNase I to remove DNA. RNA was eluted twice with 40 μL of elution buffer, heat-denatured at 65°C, and stored at -20°C overnight. RNA quality was assessed using a Nanodrop 2000 spectrophotometer (Thermo Fisher Scientific, Waltham, MA, USA), with acceptable ratios of 1.8-2.0 for 260/280 nm and 260/230 nm.

cDNA synthesis was carried out using the iScript™ Reverse Transcription Supermix (BIO-RAD, Hercules, CA, USA). RNA samples (32 μL) were mixed with 8 μL of iScript RT supermix and incubated at 25°C for 5 minutes, 46°C for 20 minutes, and then heated at 95°C for 1 minute to inactivate the reverse transcriptase. The cDNA was stored at -20°C. The concentration and quality of cDNA were also assessed using the Nanodrop 2000 spectrophotometer. For gene expression analysis, real-time RT-qPCR was performed using two 96-well plates to accommodate the 120 samples. The iTaq™ Universal SYBR^®^ Green Supermix (BIO-RAD, Hercules, CA, USA) was used for PCR reactions. Gene-specific primers were obtained from Primer Bank or custom-synthesized and reconstituted. The PCR conditions included an initial denaturation at 95°C for 2 minutes, followed by 50 cycles of denaturation at 95°C for 10 seconds, annealing/extension at 60°C for 30 seconds, and a melt curve analysis from 65 to 97°C.

Calibrated normalized relative quantification (CNRQ) of gene expression was determined using qBase+ v3.2 software (Biogazelle, Gent, Belgium). In total, 99 IRG were measured, and reference genes were selected based on the methodology described by Vandesompele and colleagues (36). The primers used, along with their specifications, were detailed in **Supplementary File S4**.

### 2.4 Plasmatic immune-related multiplex analyte profiling

Multiplex analyte profiling (xMAP^®^) was conducted on plasma samples from 20 STS patients and 20 HD controls. IRSF were analyzed using four pre-configured panels of target analytes, including one panel for general immune monitoring (Human Immune Monitoring 65-plex ProcartaPlex Panel) and three panels dedicated to immune checkpoint molecules (Human Immuno-Oncology Checkpoint 14-Plex ProcartaPlex Panel 1, Panel 2, and the 10-Plex ProcartaPlex Panel 3). The analysis was performed in accordance with the manufacturer’s instructions, and the Luminex xMAP^®^ (100/200™ system) was used to quantify the soluble proteins present in plasma samples. The data obtained from the analysis was processed using the ProcartaPlex™ Analysis App (https://apps.thermofisher.com/apps/procartaplex). Analytes with concentrations below or above the limit of detection were excluded from the analysis, resulting in a total of 81 analytes being included in the final analysis. Details of the target analytes in each immunoassay kit are provided in **Supplementary File S5**.

### 2.5 Bioinformatic tools

Principal component analysis (PCA) and unsupervised clustering analysis were conducted using the ClustVis software, accessed online at https://biit.cs.ut.ee/clustvis (37). All analyses, from data normalization to final outcomes, were performed entirely within this online tool. Initially, the data were normalized using the ln (x + 1) transformation to ensure proper distribution. PCA prediction ellipses were applied to differentiate between patient groups annotated for histological classification, treatment/response, and therapy, with unit variance scaling used for rows. Principal components (PC) were calculated using single value decomposition (SVD) with imputation. The prediction ellipses indicate the 0.95 probability range for new observations within the same group. Unsupervised clustering analysis was performed to categorize patients based on selected immune-related factors. For this analysis, rows were centered, and unit variance scaling was applied to ensure consistency in data distribution. Missing values were estimated using imputation methods. The clustering of rows was conducted using Euclidean distance as the metric, paired with Ward linkage to optimize the clustering hierarchy. Similarly, columns were clustered using a correlation distance metric combined with Ward linkage to assess the relationships between the immune factors.

The identified IRG and IRSF in each cluster for rows were further submitted to normal gene set analysis using the online software STRING version 11.5 (https://string-db.org) to construct protein-protein interaction (PPI) networks (38). The PPI network enrichment was measured, and the gene ontology (GO) pathway enrichment analysis was assessed (count in network, strength, and FDR).

### 2.6 Statistical analysis

For comparison of multiple variables, it was used the GraphPad Prism version 9.0.2 for macOS (GraphPad Software, San Diego, CA, USA). Data normalization was performed with arcsinh transformation for flow cytometry data and log10 transformation for RT-qPCR and xMAP^®^ data. Non-normally distributed variables between two groups (STS patients vs. HD control) were analyzed using multiple Mann-Whitney U tests, with false discovery rate (FDR) control and Bonferroni-Dunn correction applied. For more than two groups (e.g., histological classification, treatment/response, therapy), two-way ANOVA with FDR control followed by Bonferroni’s post-test was employed. Significance levels were set at *p* < 0.05, q = 0.05, and α = 0.05. Original values for median and inter-quartile range (IQR) were used for graphical and descriptive data.

Spearman’s correlation coefficient, calculated using GraphPad Prism version 9.0.2 for macOS (GraphPad Software, San Diego, CA, USA), was used to assess correlations between immune-related factors (immune cells, IRG, or IRSF), with significance set at *p* < 0.05.

Time-to-event survival analyses were conducted using IBM SPSS Statistics for Mac OS 26.0 (IBM Corp, Armonk, NY, USA). Cox regression and/or log-rank tests were used to evaluate the impact of studied parameters on patient survival. The variable time was defined as the time after collection (TAC), from the collection date until death or the study’s end. In some cases, time after diagnosis (TAD) was tested. Given the rarity of these tumors, using TAC instead of TAD allowed us to include more patients and gather sufficient data. To address variability in collection dates and potential bias, our analysis systematically integrated collection times into the study design. Patients who died from other causes (4 patients) were excluded. For individual variables, Cox regression analysis with hazard ratios and log-rank tests using dichotomous variables based on median values were performed. For multiple variables, standardization and multicollinearity assessments were conducted, followed by multivariate Cox analysis using Enter or Stepwise Forward Conditional methods. The proportional hazards assumption was checked using interaction terms with the log of time. Kaplan-Meier curves and log-rank tests were used to analyze the impact of peripheral immunotypes on patient survival, with multivariate Cox analysis performed as previously described.

## 3 Results

In this study an extensive analysis of immune-related factors was conducted in peripheral blood samples from 55 STS patients and age-matched HD controls. Immunophenotyping of peripheral blood was conducted by flow cytometry to assess the frequency of 83 immune cell populations, including major and minor myeloid and lymphoid cell populations, along with receptors involved in maturation, activation, and suppression. Gene expression analysis using real-time RT-qPCR was employed to evaluate the relative quantity of 99 IRG associated with immune response, as suppression, activation, and cytotoxicity. Similarly, 81 IRSF were quantified in plasma samples collected from 20 STS patients and 20 HD controls by xMAP^®^ technology using standard commercial panels.

Moreover, STS patients were studied according to histological classification, treatment/response, and therapy regimen to discuss how these clinical parameters may influence the alterations observed in STS patients comparatively with HD controls. For IRSF, due to the low sample size, statistical analysis according to histological classification and therapy were excluded, and for treatment and response evaluation, only SD and PD patients were included.

Each set of analysis of immune cells, IRG and IRSF, was performed on the same sample collected from each STS patient, allowing the construction of peripheral immune profiles based on the combined data. Afterwards, unsupervised clustering analysis was performed to identify similar immune profiles within patients and classify patients according to their immunotype. The immunotype classification was inspected for its impact on patient survival in order to explore the potential of peripheral blood samples as a tool for STS monitoring.

### 3.1 Contraction of B cell and CD4 T cell compartments in STS patients

The immunophenotyping was performed on peripheral blood samples collected from STS patients and HD controls. Using the automated hematological counter, the leucocyte absolute frequency and the GR, MO, and LY absolute and relative frequencies were assessed. It was observed a significant decrease in LY absolute and relative frequency (0.9 cells/<L, IQR: 0.6–1.4, N = 49; 16.6% of LEU, IQR: 8.4–27.6, N = 49; respectively) when compared with HD controls (2.1 cells/<L, IQR: 1.7–2.5, N = 45, adjusted *p* (adj *p*) < 0.000001; 32.9% of LEU, IQR: 24.5–39.6, N = 45, adj *p* = 0.000002) (**Supplementary Files S6A, B**). Alongside, it was observed an increase in the relative frequency of GR in STS patients (74.3% of LEU, IQR: 57.8–82.5, N = 49) compared with HD controls (61.1% of LEU, IQR: 52.9–67.6, N = 45, adj *p* = 0.011569) (**Supplementary file S6B**). No significant alterations were observed for both absolute and relative frequencies of MO (**Supplementary Files S6A, B**). Using flow cytometry, the relative frequency of DC and MDSC was assessed, and the absolute frequency of both was estimated based on the LEU absolute count. Yet no significant alterations were observed (**Supplementary Files S6A, B**). Similarly, no differences were observed when analyzing the groups of patients according to the clinical parameters (data not shown).

**Figure 1:**
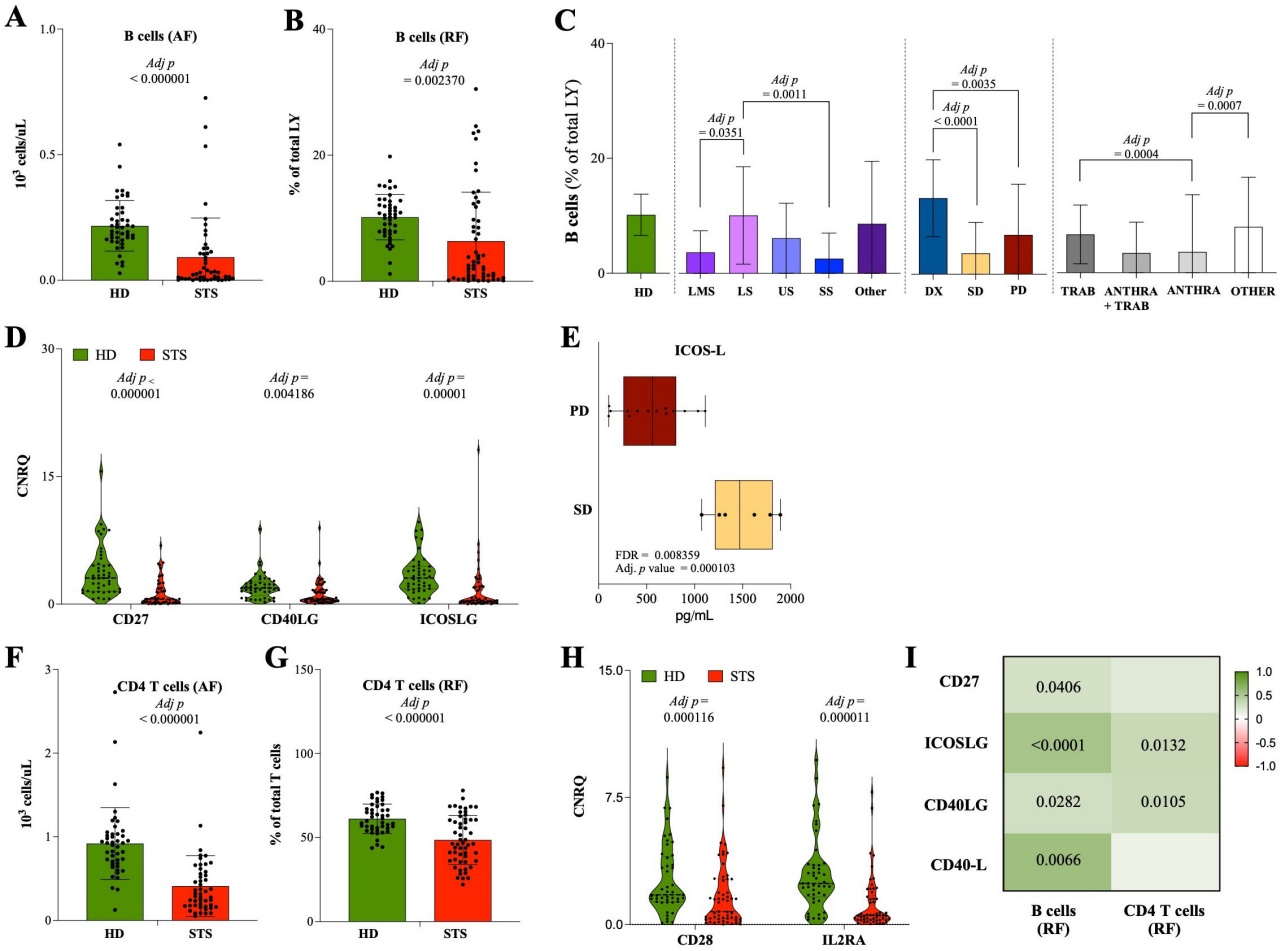
Contraction of B cell and CD4 T cell compartments in STS patients. Multiparametric flow cytometry and real-time RT-qPCR were used to analyze immune cells and IRG in peripheral whole blood samples, and xMAP technology was used to analyze IRSF in plasma samples. **(A)** Absolute frequency of B cells observed in STS patients and HD controls. **(B)** Relative frequency of B cells observed in STS patients and HD controls. **(C)** Relative frequency of B cells according to histological classification, treatment/response, and therapy. **(D)** Relative quantification of the IRG CD27, ICOSLG, and CD40LG observed in STS patients and HD controls. **(E)** Relative quantification of the IRSF ICOS-L according to treatment/response. **(F)** Absolute frequency of CD4 T cells observed in STS patients and HD controls. **(G)** Relative frequency of CD4 T cells observed in STS patients and HD controls. **(H)** Relative quantification of the IRG CD28 and IL2RA observed in STS patients and HD controls. **(I)** Correlation analysis of B cells and CD4 T cells with the IRG CD27, ICOSLG, and CD40LG, and with the IRSF CD40-L. Original values were used for data representation using Tukey method, while transformed values of immune cells (arcsin transformed), IRG (log10 transformed), and IRSF (log10 transformed) were used for statistical analysis. In A, B, D-H, it was conducted multiple Mann-Whitney U tests controlling for FDR, followed by Bonferroni-Dunn method to obtain the adjusted *p*-values. In C, it was conducted 2-way ANOVA controlling for FDR (q = 0.05), followed by Bonferroni's post-test for multiple comparisons between groups. In I, Spearman's correlation analysis was performed, and the coefficient matrix was plotted, with significant *p*-values represented. The color scale represents the direction of association, green means positive correlation and red means negative correlation. Statistical significance was set at *p* < 0.05, q = 0.05 and a = 0.05. Legend: HD, healthy donors; STS, soft tissue sarcoma; LMS, leiomyosarcoma; LS, liposarcoma; US, undifferentiated sarcoma; SS, synovial sarcoma; DX, patients indicated for surgery; SD, stable disease; PD, progression disease; TRAB, trabectedin-base chemotherapy; ANTHRA, anthracycline-based therapy; ANTHRA + TRAB, trabectedin-based therapy after anthracycline-based therapy; AF, absolute frequency; RF, relative frequency; IRG, immune-related genes; IRSF, immune-related soluble factors; CNRQ, calibrated normalized relative quantity; FDR, false discovery rate; Adj p, adjusted p-value.

It was also considered the frequency of LY subpopulations. The comparative analysis between STS patients and HD controls revealed that STS patients have significantly lower B cell absolute and relative frequency (0.02 cells/<L, IQR: 0.01–0.11, N = 49; 2.5% of LY, IQR: 0.5–9.8, N = 55; respectively) when compared with HD controls (0.19 cells/<L, IQR: 0.16–0.27, N = 45, adj *p* < 0.000001; 10.5% of total LY, IQR: 7.8–12.4, N = 45, adj *p* = 0.002370) (**Figures 1A, B**). The absolute frequency of T cells (0.69 cells/<L, IQR: 0.39–1.06, N = 49) and NK cells (0.08 cells/<L, IQR: 0.05–0.16, N = 49) was also significantly decreased in STS patients when compared with HD controls (1.38 cells/<L, IQR: 1.13–1.34, N = 45, adj *p* = 0.000004; 0.26 cells/<L, IQR: 0.16–0.4, N = 45, adj *p* < 0.000001; respectively), whereas no significant differences were observed for relative frequencies (**Supplementary Files S7A, B**). No significant differences were observed for the absolute or relative frequency of NKT-like cells (**Supplementary Files S7A, B**).

Considering the clinical parameters, significant differences were observed in the relative frequency of B cells. According to histological classification, LS patients exhibited a significantly higher frequency of B cells (8.5% of LY, IQR: 2.5–20.1, N = 9) when compared with LMS (2.1% of LY, IQR: 0.8–7.8, N = 18, adj *p* = 0.0351) and SS patients (0.7% of LY, IQR: 0.2–3.4, N = 8, adj *p* = 0.0011) (**Figure 1C**). Regarding treatment and response, a significant lower relative frequency of B cells was observed in SD (1.1% of LY, IQR: 0.4–5.1, N = 26, adj *p* < 0.0001) and PD (2.3% of LY, IQR: 1.1–10.9, N = 21, adj *p* = 0.0035) patients compared with DX patients (13.5% of LY, IQR: 10.3–18.4, N = 8) (**Figure 1C**). Lastly, when analyzing the therapy regimen, it was observed that there were significantly lower levels of B cells in ANTHRA patients (0.14% of LY, IQR: 0.09–0.74, N = 9) than in the TRAB (6.1% of LY, IQR: 2.6–12.1, N = 8, adj *p* = 0.0004) or OTHER (6.7% of LY, IQR: 0.5–12.3, N = 11, adj *p* = 0.0007) group of patients (**Figure 1C**).

In whole blood gene expression analysis, lower expression levels of CD27, CD40LG, and ICOSLG (0.586 CNRQ, IQR: 0.208–1.843, N = 55; 0.600 CNRQ, IQR: 0.364–1.651, N = 55; 0.400 CNRQ, IQR: 0.209–2.952, N = 49; respectively) were observed in STS patients comparatively with HD controls (3.059 CNRQ, IQR: 1.497–4.649, N = 45, adj *p* < 0.000001; 1.899 CNRQ, IQR: 1.018–2.608, N = 45, adj *p* = 0.004186; 3.060 CNRQ, IQR: 1.776–4.430, N = 45, adj *p* = 0.00001; respectively) (**Figure 1D**). Considering the clinical parameters evaluated, no significant differences were observed (data not shown), yet PD patients exhibit a tendency for a decrease in the plasma levels of ICOS-L (561.9 pg/mL, IQR: 258.6–808.8, N = 20) compared with SD patients (1470.3 pg/mL, IQR: 1211.6–1813.1, N = 6, FDR = 0.008359; adj *p* = 0.000103) (**Figure 1E**).

Moreover, besides no alterations in the relative frequency of T cells (**Supplementary File S7B**), it was observed a significant reduction in the absolute and relative frequency of CD4 T cells (0.32 cells/<L, IQR: 0.17–0.57, N = 49; 46.3% of T cells, IQR: 36.2–60.6, N = 55; respectively) in STS patients comparatively with HD controls (0.9 cells/<L, IQR: 0.68–1.03, N = 45, adj *p* < 0.000001; 59.8% of T cells, IQR: 55.3–67.8, N = 45, *p* = 0.000963; respectively) (**Figures 1F, G**; **Supplementary File S8**). The gene expression analysis revealed decreased expression levels of CD28 and IL2RA in STS patients (0.770 CNRQ, IQR: 0.331–2.681, N = 55; 0.583 CNRQ, IQR: 0.277–1.709, N = 54) comparatively with HD controls (3.059 CNRQ, IQR: 1.497–4.639, N = 45, adj *p* = 0.000116; 2.429, IQR: 1.638–3.510, N = 45, adj *p* = 0.000011; respectively) (**Figure 1H**).

Furthermore, the correlation analysis of B cells and CD4 T cells with the IRG CD27, CD40LG, and ICOSLG in STS patients showed a significant positive correlation between the relative frequency of B cells and the gene expression levels of the IRG CD27 (Spearman r = 0.277, *p* = 0.0406), ICOSLG (Spearman r = 0.541, *p* < 0.0001), and CD40LG (Spearman r = 0.296, *p* = 0.0282), and the plasma levels of CD40-L (Spearman r = 0.586, *p* = 0.0066). Similarly, the relative frequency of CD4 T cells was found to be positively correlated with the gene expression levels of ICOSLG (Spearman r = 0.352, *p* = 0.0132) and CD40LG (Spearman r = 0.342, *p* = 0.0105), with statistical value (**Figure 1I**).

### 3.2 The major impact of immunosuppression (MDSC and Treg) in STS patients

**Figure 2:**
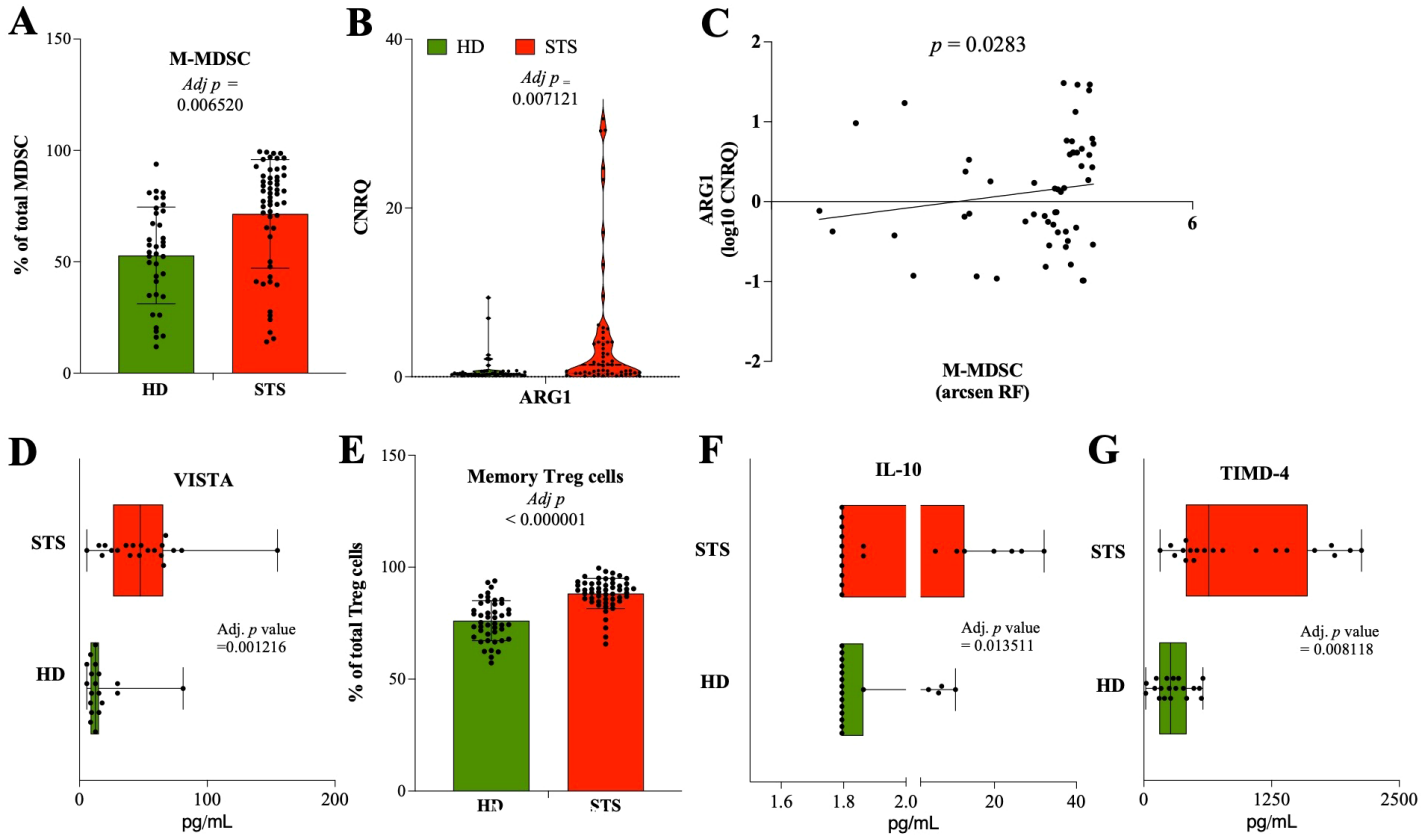
Major impact of immunosuppression (MDSC and Treg) in STS patients. Multiparametric flow cytometry and real-time RT-qPCR were used to analyze immune cells and IRG in peripheral whole blood samples, and xMAP technology was used to analyze IRSF in plasma samples. **(A)** Relative frequency of M-MDSC cells observed in STS patients and HD controls. **(B)** Relative quantification of the IRG ARG1 observed in STS patients and HD controls. **(C)** Correlation analysis of M-MDSC with the IRG ARG1. **(D)** Relative quantification of the IRSF TIMD-4 observed in STS patients and HD controls. **(E)** Relative frequency of Treg cells observed in STS patients and HD controls. **(F)** Relative quantification of the IRSF IL-10 observed in STS patients and HD controls. **(G)** Relative quantification of the IRG TIMD-4 observed in STS patients and HD controls. Original values were used for data representation using Tukey method, while transformed values of immune cells (arcsin transformed), IRG (log10 transformed), and IRSF (log10 transformed) were used for statistical analysis. In **(A, B, D-G)**, it was conducted multiple Mann-Whitney U tests controlling for FDR, followed by Bonferroni-Dunn method to obtain the adjusted *p*-values. In **(C)**, Spearman’s correlation analysis was performed. Statistical significance was set at *p* < 0.05, q = 0.05 and α = 0.05. HD, healthy donors; STS, soft tissue sarcoma; MDSC, myeloid-derived suppressor cells; M-MDSC, monocytic-MDSC; Treg, regulatory T cells; IRG, immune-related genes; IRSF, immune-related soluble factors; CNRQ, calibrated normalized relative quantity; FDR, false discovery rate; Adj p, adjusted p-value.

In this cohort of STS patients, a significant expansion of circulating monocytic-MDSC (M-MDSC) was observed (79.7% of MDSC, IQR: 58.5–88.6, N = 54) when compared with the HD controls (54.3% of MDSC, IQR: 35.1–72.3, N = 35, adj *p* = 0.00652) (**Figure 2A**). The gene expression levels of the IRG ARG1 were also found to be significantly increased in STS patients’ peripheral whole blood (1.419, IQR: 0.420–4.124, N = 55), compared with HD controls (0.335 CNRQ, IQR: 0.256–0.658, N = 43, adj *p* = 007121) (**Figure 2B**). Moreover, the relative frequency of circulating M-MDSC and the gene expression levels of ARG1 were to be found positively correlated in STS patients, with a statistical value (Spearman r = 0.2985, *p* = 0.0283) (**Figure 2C**). Using xMAP technology, the quantification of soluble VISTA in plasma samples was found to be superior in STS patients (47.6 pg/mL, IQR: 26.3–65.5, N = 20) than in HD controls (12.5 pg/mL, IQR: 8.6–15.2, N = 19, adj *p* = 0.001216) (**Figure 2D**).

The maturation state of CD4 T cells was evaluated, and it was observed that there was a decreased relative frequency of *naïve* CD4 T cells (9.4% of CD4 T cells, IQR: 5.2–21.8, N = 55) in STS patients than in HD controls (34.3% of CD4 T cells, IQR: 17.1–43, N = 45, adj *p* < 0.000001) (**Supplementary File S9A**). The relative frequency of Th1, Th2, Th17, and Treg cells was also evaluated. Significantly lower frequencies of Th2 cells (42.2% of CD4 T cells, IQR: 25.9–52.6, N = 55) and increased frequencies of Th17 cells (13.2% of CD4 T cells, IQR: 10.1–19, N = 55) were observed in STS patients when compared with HD controls (53.1% of CD4 T cells, IQR: 46.4–63.6, N = 45, adj *p* = 001292; 7.2% of CD4 T cells; IQR: 6.1–11.6, N = 45, adj *p* = 000194; respectively), while no differences were observed for Th1 nor Treg cells (**Supplementary File S9B**). On the other hand, the frequency of memory Treg cells was found increased in the peripheral blood of STS patients (89.7% of Treg cells, IQR: 85.3–92.8, N = 53) compared with HD controls (75.3% of Treg cells, IQR: 69.5–83.7, N = 75.3, adj *p* < 0.000001) (**Figure 2E**). The analysis of plasma samples also revealed a higher significant quantification of IL-10 in STS patients (1.8 pg/mL, IQR: 1.8–12.7, N = 19) than in HD controls (1.8 pg/mL, IQR: 1.8–1.9, N = 19, adj *p* = 0.013511) (**Figure 2F**). Moreover, the analysis of the IRSF revealed increased levels of TIMD-4 in plasma samples from STS patients (635.9 pg/mL, IQR: 411.1–1603.8, N = 20) compared with HD controls (261.5 pg/mL, IQR: 150.5–418.9, N = 19, adj *p* = 0.008118) (**Figure 2G**).

### 3.3 Compromised cytotoxic potential associated with CD56^dim^ NK cells and CD8 T cells in STS patients

**Figure 3:**
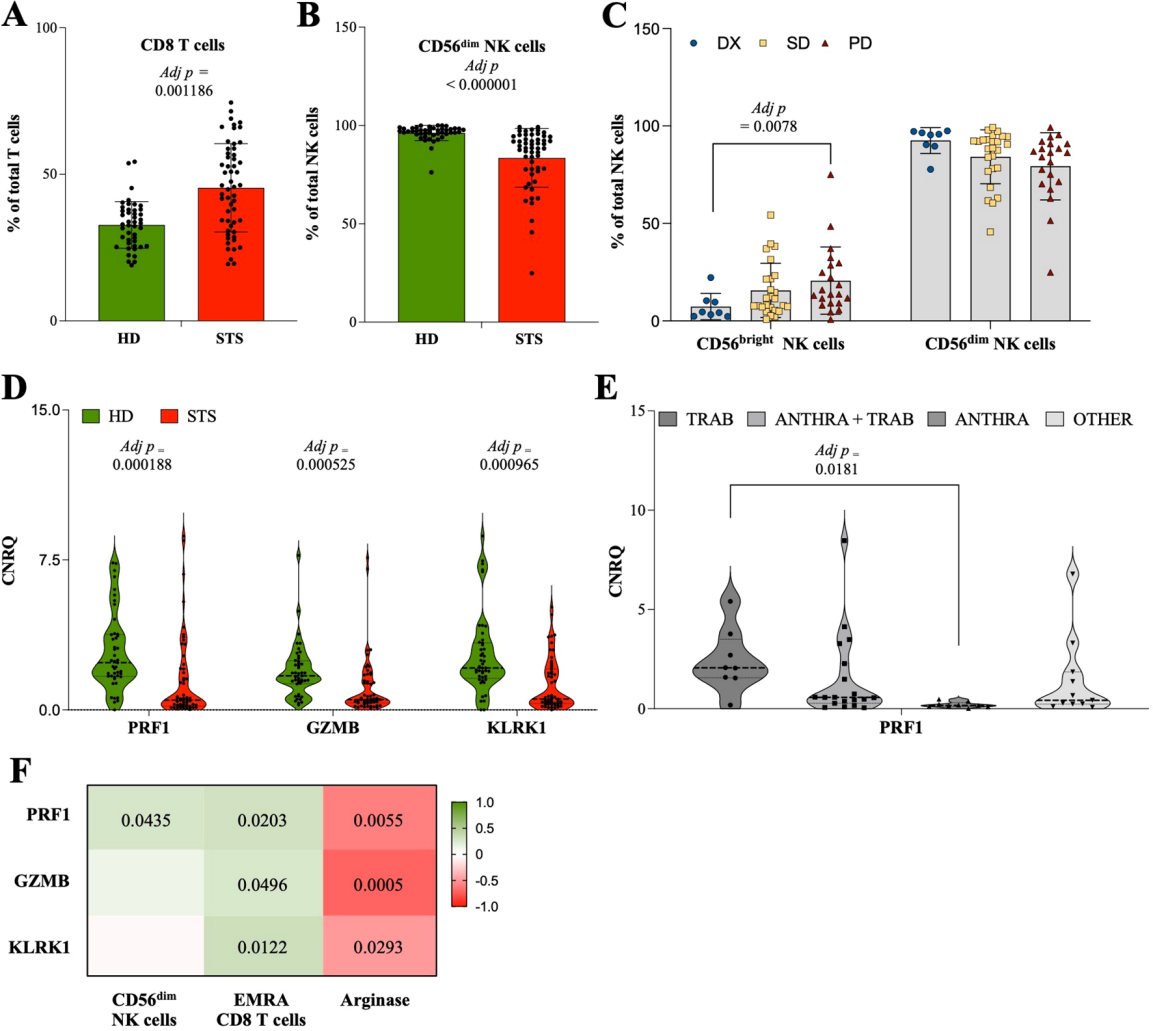
Compromised cytotoxic potential associated with CD56^dim^ NK cells and CD8 T cells in STS patients. Multiparametric flow cytometry and real-time RT-qPCR were used to analyze immune cells and IRG in peripheral whole blood samples. **(A)** Relative frequency of CD8 T cells observed in STS patients and HD controls. **(B)** Relative frequency of CD56^dim^ NK cells observed in STS patients and HD controls. **(C)** Relative frequency of CD56^dim^ NK cells according to treatment/response. **(D)** Relative quantification of the IRG PRF1, GZMB, and KLRK1 observed in STS patients and HD controls. **(E)** Relative quantification of the IRG PRF1 according to therapy. **(F)** Correlation analysis of CD56^dim^ NK cells, EMRA CD8 T cells, and the IRSF arginase with the IRG PRF1, GZMB, and KLRK1. Original values were used for data representation using Tukey method, while transformed values of immune cells (arcsin transformed) and IRG (log10 transformed) were used for statistical analysis. In **(A, B, D)** it was conducted multiple Mann-Whitney U tests controlling for FDR, followed by Bonferroni-Dunn method to obtain the adjusted *p*-values. In **(C, E)**, it was conducted 2-way ANOVA controlling for FDR (q = 0.05), followed by Bonferroni’s post-test for multiple comparisons between groups. In F, Spearman’s correlation analysis was performed, and the coefficient matrix was plotted, with significant *p*-values represented. The color scale represents the direction of association, green means positive correlation and red means negative correlation. Statistical significance was set at *p* < 0.05, q = 0.05 and α = 0.05. HD, healthy donors; STS, soft tissue sarcoma; DX, patients indicated for surgery; SD, stable disease; PD, progression disease; TRAB, trabectedin-base chemotherapy; ANTHRA, anthracycline-based therapy; ANTHRA + TRAB, trabectedin-based therapy after anthracycline-based therapy; IRG, immune-related genes; CNRQ, calibrated normalized relative quantity; FDR, false discovery rate; Adj p, adjusted p-value.

The analysis of the peripheral blood and plasma samples showed a significant increase in the relative frequency of CD8 T cells (45.7% of T cells, IQR: 32.4–57.1, N = 55) in STS patients when compared with HD controls (32.9% of T cells, IQR: 26–37.8, N = 45, adj *p* = 0.001186) (**Figure 3A**), while no differences were observed for absolute frequency (**Supplementary File S8A**). Similarly, no alterations were observed for CD8 T cell subpopulations according to maturation states (**Supplementary File S9C**). Moreover, the relative frequency of CD56^dim^ NK cells was found to be significantly lower in STS patients (88.5% of NK cells, IQR: 77.7–94.3, N = 55) than in HD controls (97% of NK cells, IQR: 95.6–98.4, N = 45, adj *p* < 0.000001) (**Figure 3B**). The analysis according to treatment/response revealed significant increased levels of CD56^bright^ NK cells in PD patients (13.7% of NK cells, IQR: 9.1–29.2, N = 21) in comparison with DX patients (4.4% of NK cells, IQR: 2.6–10.2, N = 8, *p* = 0.0078) (**Figure 3C**). Gene expression analysis of whole blood samples showed that STS patients had significantly decreased levels of the IRG PRF1 (0.484, IQR: 0.185–2.049, N = 55), GZMB (0.515 CNRQ, IQR: 0.351–1.457, N = 55), and KLRK1 (0.545 CNRQ, IQR: 0.318–2.047, N = 55) comparatively to HD controls (2.365 CNRQ, IQR: 1.675–3.772, N = 45, adj *p* = 0.000188; 1.705, IQR: 1.312–2.453, N = 45, adj *p* = 0.000525; 2.098 CNRQ, IQR: 1.580–3.354, N = 45, adj *p* = 0.000965; respectively) (**Figure 3D**). It was also shown that ANTHRA patients exhibited lower levels of PRF1 (0.168 CNRQ, IQR: 0.111–0.306, N = 9), which were significantly decreased comparatively with TRAB patients (2.060 CNRQ, IQR: 1.560–3.500, N = 8, adj *p* = 0.0181) (**Figure 3E**). Additionally, through the correlation analysis of CD8 T cells and CD56^dim^ NK cells with the gene expression levels of PRF1, GZMB, and KLRK1 in STS patients, it was observed a significant positive correlation of CD56^dim^ NK cells with PRF1 (Spearman r = 0.273, *p* = 0.0435) and EMRA CD8 T cells with PRF1 (Spearman r = 0.312, *p* = 0.0203), GZMB (Spearman r = 0.266, *p* = 0.0496), and KLRD1 (Spearman r = 0.336, *p* = 0.0122) (**Figure 3F**). Contrarily, the plasma level of Arginase in STS patients (699.6 pg/mL, IQR: 91.5–3417, N = 20) was found to be negatively correlated, with statistical value, with PRF1 (Spearman r = -0.597, *p* = 0.0055), GZMB (Spearman r = -0.708, *p* = 0.0005), and KLRK1 (Spearman r = -0.487, *p* = 0.0293) (**Figure 3F**).

### 3.4 Immunotype classification and impact on patient survival

**Figure 4:**
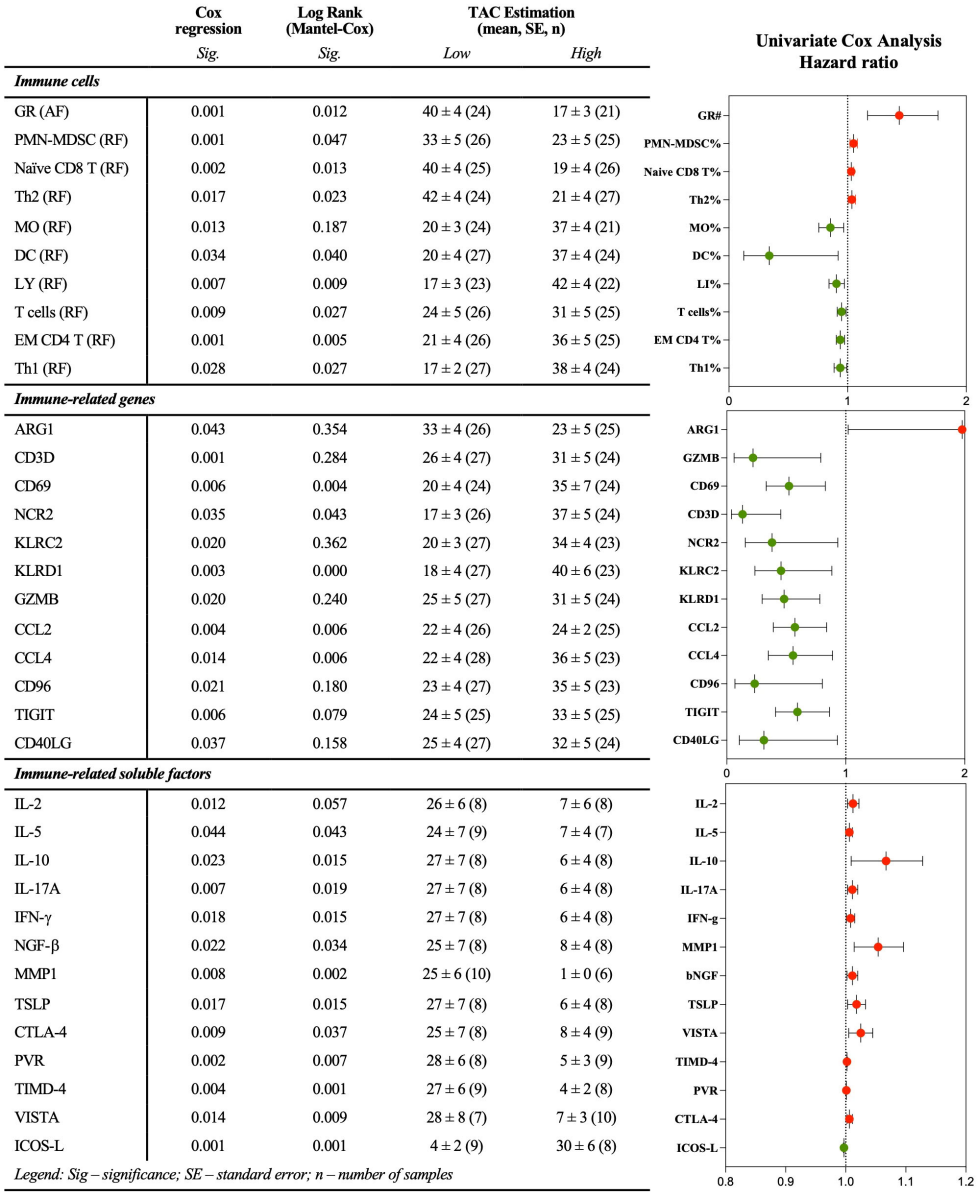
Immune-related factors individually associated with patient survival. Multiparametric flow cytometry and real-time RT-qPCR were used to analyze immune cells and IRG in peripheral whole blood samples, and xMAP technology was used to analyze IRSF in plasma samples. Univariate Cox analysis, log-rank test, and graphical representation of the survival-associated hazard ratio. Red and green dots represent factors associated with risk and protection, respectively. Statistical significance was set at p < 0.05. GR, granulocytes; AF, absolute frequency; RF, frequency; MO, monocytes; DC, dendritic cells; MDSC, myeloid-derived suppressor cells; PMN-MDSC, polymorphonuclear-MDSC; LY, lymphocytes; DN, double negative; EM, effector memory; Th, T helper.; IRG, immune-related genes; IRSF, immune-related soluble factors.

Furthermore, it was aimed at integrating the data obtained from flow cytometry, real-time RT-qPCR, and xMAP analysis to construct peripheral immune profiles and explore their value for monitoring STS patients. To achieve that, and considering the large number of variables, immune cells, IRG, and IRSF, first it was investigated the potential impact of each variable on patient survival, defined as time-to-death event counting from the time of the sample collection to the event of death or the end of the study (May 2023), denominated as time after collection (TAC). Through Cox regression and log-rank tests with an appreciation of KM curves, it was identified immune-related factors significantly correlated with patient survival. The calculated hazard ratio associated with the Cox regression analysis indicated the level of risk or protection associated with each variable. Detailed statistics are depicted in **Figure 4**. Within the immune cell populations analyzed, increased levels of GR (AF), polymorphonuclear-MDSC (PMN-MDSC), Th2, and *naïve* CD8 T cells were indicated as risk factors, whereas increased levels of MO, DC, LY, T cells, EM CD4 T, and Th1 cells were indicated as protection factors (**Figure 4**). Among IRG, heightened levels of ARG1 were linked to increased risk, while increased levels of GZMB, CD69, CD3D, NCR2, KLRD1, CCL2, CCL4, CD96, TIGIT, and CD40LG were associated with protection (**Figure 4**). In the analysis of IRSF, factors such as IL-2, IL-5, IL-10, IL-17A, IFN-γ, MMP1, bNGF, TLSP, VISTA, TIMD-4, PVR, and CTLA-4 were indicated as risk factors, while an increased level of soluble ICOS-L was associated with protection in STS patients (**Figure 4**).

It was also performed a multivariable Cox proportional hazards regression analysis to assess the impact of immune-related variables on patient survival, adjusting for commonly known prognostic factors such as tumor site (extremity, trunk non-RPS, RPS, gynecologic, head & neck), tumor grade (low grade, high grade, metastatic primary, metastatic recurrent), age at diagnosis, and age at collection time. Due to high multicollinearity and the sample size relative to the number of variables, a Stepwise Method with Forward selection (Likelihood Ratio) was employed. Data was standardized, and the proportional hazards assumption was checked using interaction terms with the log of time. The results indicated that gene expression of CD40LG (*p* = 0.001), and the combination of CD40LG and the relative frequency of PMN-MDSC (*p* = 0.022), are significant predictors of patient survival (**Supplementary File S10**). The proportional hazards assumption was validated for both CD40LG and PMN-MDSC predictors (*p* > 0.05).

Next, a database containing individual parameters associated with either risk or protection (comprising 35 immune-related variables) for each of the 55 STS patients included in the study was constructed and then uploaded into the ClustVis web platform (http://biit.cs.ut.ee/clustvis/) for data visualization. The data was processed using PCA prediction ellipses and heatmaps (37). For data normalization and to reduce skewness, the original values were transformed (ln (x + 1)), and row centering and unit variance scaling were applied to enhance comparability across different immune-related factors. Additionally, imputation methods were employed to handle missing values, ensuring accurate estimations. Unit variance scaling was applied to rows and SVD with imputation was used to calculate PC.

**Figure 5:**
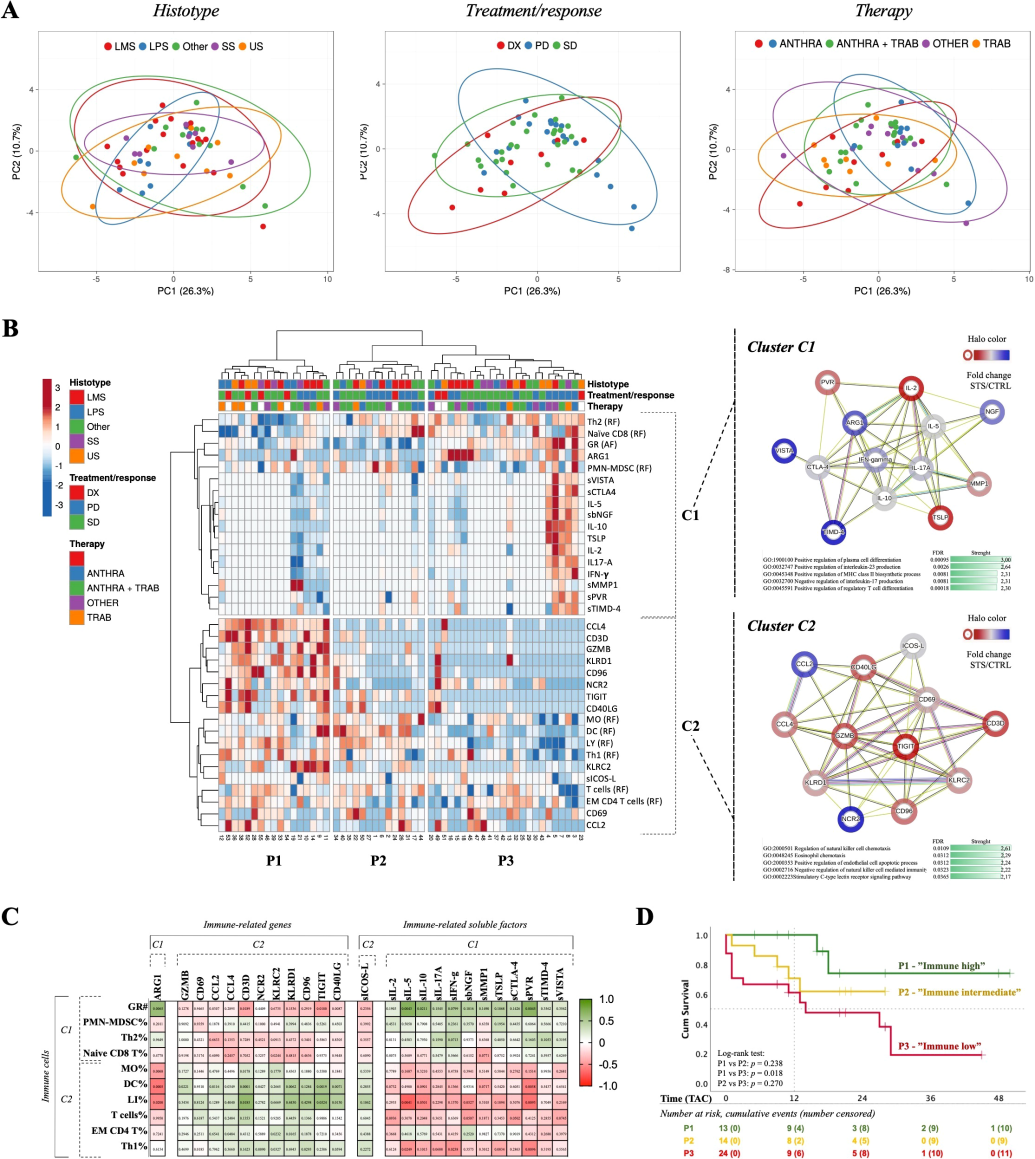
Immunotype classification and impact on patient survival. Multiparametric flow cytometry and real-time RT-qPCR were used to analyze immune cells and IRG in peripheral whole blood samples, and xMAP technology was used to analyze IRSF in plasma samples. **(A)** PCA according to histological classification, treatment/response, and therapy. Unit variance scaling was applied to rows and SVD with imputation was used to calculate principal components. Prediction ellipses are such that with a 0.95 probability, a new observation from the same group will fall inside the ellipse. **(B)** Unsupervised clustering analysis of the selected immune cells, IRG and IRSF, and PPI network of IRG and IRSF identified in both C1 and C2 clusters. Heatmap to visualize clustering of multivariate data for 55 STS patients. ClustVis was accessed online (https://biit.cs.ut.ee/clustvis) and patients were plotted by columns while the selected parameters were plotted by rows. Three clusters of patients (P1, P2 and P3) and two clusters of immune-related factors (C1 and C2) were identified. PPI and cluster analysis of the immune-related factors present in each cluster of the heatmap constructed for the 55 STS patients, using the online software STRING (version 11.5). C1 cluster, 13 nodes and 45 edges. The PPI network enrichment was found to be statistically significant (*p* < 1.0e^-16^). C2 cluster, 12 nodes and 45 edges. The PPI network enrichment was found to be statistically significant (*p* < 1.0e^-16^). **(C)** Spearman’s correlation analysis of immune cells clustered in C1 and C2 with IRG and IRSF. The coefficient matrix was plotted with the *p*-values represented. The color scale represents the direction of association, green means positive correlation and red means negative correlation. **(D)** Survival analysis based on peripheral immunotypes. Kaplan-Meier curves generated from a cohort of 55 STS patients, categorized into P1 (“immune high”), P2 (“immune intermediate”), and P3 (“immune low”) immunotypes. Censored events were identified as a cross in the respective curves. The number of patients at risk are represented in the table below the graph. Statistical significance was set at *p* < 0.05. PCA, principal component analysis; PC, principal component; IRG, immune-related genes; IRSF, immune-related soluble factors; LMS, leiomyosarcoma; LS, liposarcoma; US, Undifferentiated sarcoma; SS, synovial sarcoma. ANTHRA, Anthracycline-based chemotherapy; TRAB, trabectedin-based therapy; Naïve, patients indicated for surgery; PPI, protein-protein interaction; IRG, immune-related genes.; IRSF, immune-related soluble factors; UC, upper cluster; LC, lower cluster; GO, gene ontology; FDR, false discovery rate; TAC, time after collection.

First, histological classification, treatment/response, and therapy were considered for creating PCA prediction ellipses (**Figure 5A**). The degree of a similarity or dissimilarity between the groups might be deduced by looking at the placement and overlap of the ellipses. The X and Y axes are represented by PC1 and PC2, which explain 26.3% and 10.7% of the total variation, respectively. The overlap observed in the prediction ellipses indicated an independence of peripheral immunotypes from histological classification, treatment/response, and therapy. Following this, unsupervised clustering analysis was conducted, resulting in the generation of a heatmap featuring 55 columns (representing patients) and 35 rows (representing immune factors) (**Figure 5B**, left). The clustering of columns (patients) utilized the correlation distance metric and Ward linkage, while the clustering of rows (immune-related factors) employed Euclidean distance and Ward linkage. The analysis revealed three major patient clusters, denoted as P1, P2, and P3, which comprised 17/55, 14/55, and 24/55 of the patients, respectively. Moreover, two clusters were identified for the rows, representing the immune-related factors: an upper cluster (C1) containing 17/35 factors and a lower cluster (C2) containing 18/35 factors.

Then, the immune-related variables that distinguish between patient groups were examined. In C1, the immune populations GR (#), PMN-MDSC (%), *Naïve* CD8 T cells (%), and Th2 cells (%) were clustered together with the IRG ARG1, and the IRSF sVISTA, sCTLA-4, IL-5, bNFG, sIL10, sTLSP, L-2, IL-17A, IFN-g, sMMP-1, sPVR, and sTIMD-4. In C2, the immune populations MO%, DC%, LY%, T cells %, EM CD4 T cells%, and Th1 cells% were clustered together with the IRG CD3D, CCL4, GZMB, CD96, NCR2, TIGIT, CD40LG, KLRC2, CD69, and CCL2, and the IRSF sICOS-L. Using STRING version 11.5 (https://string-db.org), a bioinformatic analysis compared the set of genes and proteins within each C1 and C2 cluster with the whole proteome to identify associated biological pathways (38). For each cluster, a table with factor names and normalized means against the HD controls was uploaded, and a PPI network analysis was constructed (**Figure 5B**, right). The PPI network enrichment value was < 1x10^-16^ for both sets of genes (C1 and C2). A functional enrichment analysis of the GO pathway was performed for each gene or protein set, and the top five significant pathways with higher strength were considered. For C1, the top five GO biological processes identified were: positive regulation of plasma cell differentiation, positive regulation of interleukin-23 production, positive regulation of MHC class II biosynthetic process, negative regulation of interleukin-17 production, and positive regulation of regulatory T cell differentiation (**Figure 5B**, right, top). For C2, the top five GO biological processes identified were: regulation of NK cell chemotaxis, eosinophil chemotaxis, positive regulation of endothelial cell apoptotic process, negative regulation of NK cell-mediated immunity, and stimulatory C-type lectin receptor signaling pathway (**Figure 5B**, right, bottom).

Moreover, individual correlations between each immune cell population and IRG and IRSF were investigated. A multivariate analysis was conducted, and a Spearman correlation matrix was plotted, depicting the degree of association between factors (Spearman’s R for each pair) (**Figure 5C**). The colored scale in the matrix indicates the correlation direction, with green representing a positive correlation and red representing a negative correlation. Wells with values in the matrix represent significant *p*-values. Immune cells individually associated with risk (GR (#), PMN-MDSC (%), *naïve* CD8 T cells (%), Th2 (%)) exhibited a similar correlation pattern with IRG and IRSF. Conversely, immune cell populations associated with protection (MO%, DC%, LY%, T cells %, EM CD4 T cells, and Th1 cells%) demonstrated a similar pattern within each other, opposite to that observed for risk-associated populations.

Considering GO functional analysis, Spearman correlation analysis, and immunobiology knowledge, it was determined that the C1 cluster was enriched in inflammatory/immunosuppressive factors, while the C2 cluster was enriched in effector/cytotoxic factors. Therefore, the peripheral immune profiles P1, P2, and P3 were categorized as “immune high,” “immune intermediate,” and “immune low,” respectively. P1 patients displayed reduced levels of C1 factors along with elevated levels of C2 factors, contrary to the observation for P3 patients. P2 patients exhibited intermediate expression levels of C1 and C2 factors, reflecting an intermediate profile.

The implication of peripheral immunotypes on patient clinical outcomes was then investigated. Similar to the survival analysis performed for individual factors, the TAC was set from the time of blood collection until death event occurrence or the end of the study. The resulting KM curves are shown in **Figure 5D**. The P1 “immune high,” P2 “immune intermediate,” and P3 “immune low” patients exhibited an estimated TAC of 41 months (N = 13), 20 months (N = 14), and 19 months (N = 24), respectively. The survival rate at 12 months for C1 “immune high” was 100%, whereas for C2 “immune intermediate” it was 70%, and for C1 “immune low” it was about 60%. The log-rank test was employed, and significant differences were observed for the survival curves of C1 “immune high” and C3 “immune low” patients (*p*-value = 0.018). The survival analysis of immunotypes using TAD was assessed, but no significant values were found (**Supplementary File S11**).

**Figure 6:**
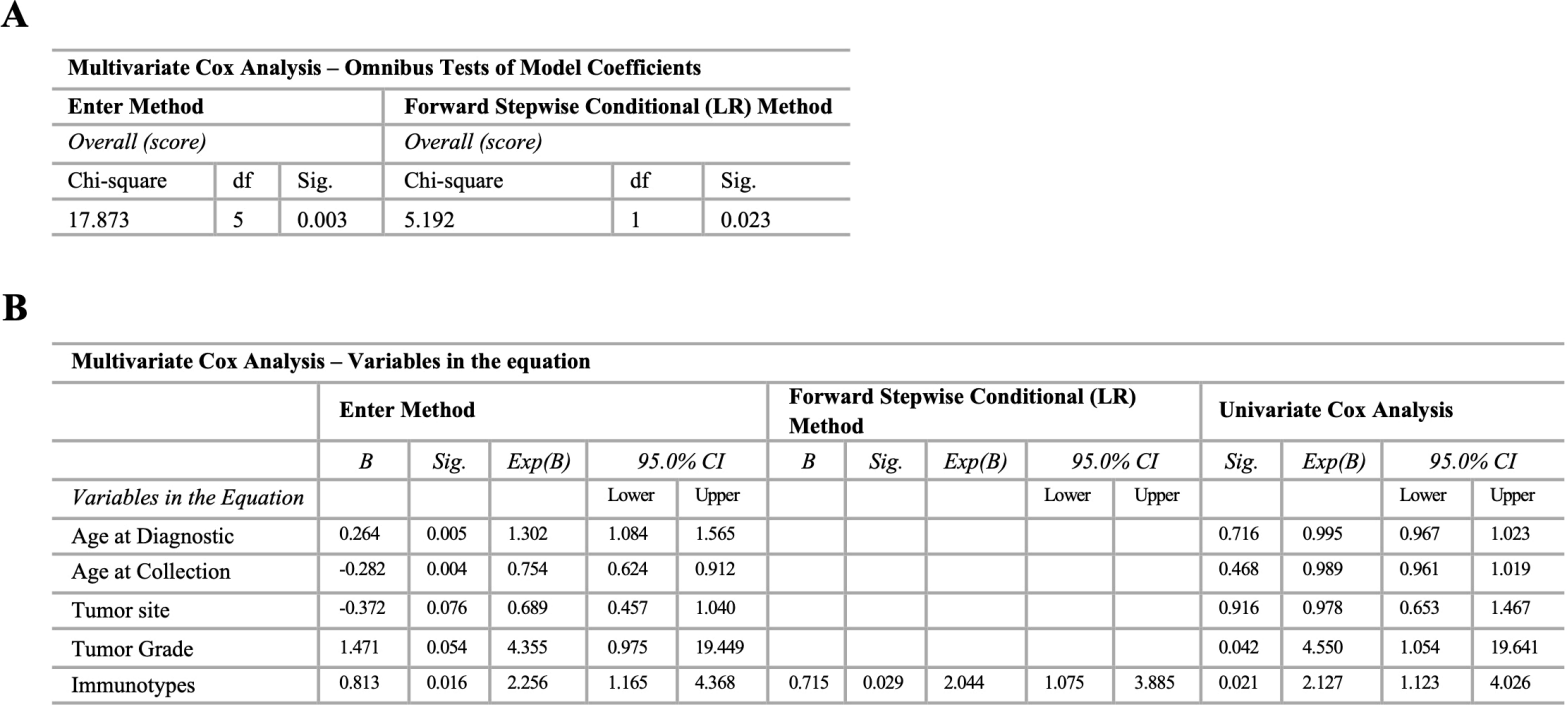
Multivariate Cox analysis of immunotypes adjusting for common prognostic factors. Multiparametric flow cytometry and real-time RT-qPCR were used to analyze immune cells and IRG in peripheral whole blood samples, and xMAP technology was used to analyze IRSF in plasma samples. Enter and stepwise method with forward selection (likelihood ratio) for immunotypes adjusting for tumor site (extremity, trunk non-RPS, RPS, gynecologic, head & neck), tumor grade (low grade (non-metastatic primary); high grade (non-metastatic primary recurrent; metastatic primary; metastatic recurrent), age at diagnosis, and age at collection time. **(A)** Overall model including all the variables. Enter and forward Stepwise Conditional (LR) method. **(B)** Variables in the equation for the Enter and forward Stepwise Conditional (LR) method, and univariate Cox analysis. Statistical significance was set at *p* < 0.05.

In line with the survival analysis of individual variables, the significance of peripheral immunotypes was assessed using a multivariate Cox regression model adjusted for tumor site, tumor grade, age at diagnosis, and age at collection time. The overall model, which incorporated all variables, significantly predicted patient survival when employing both the Enter (*p* = 0.003) and forward Stepwise (*p* = 0.023) methods (**Figure 6A**). Using the Enter method, the variable immunotypes (*p* = 0.016) retained its significance. Additionally, age at diagnosis (*p* = 0.005) and age at collection (*p* = 0.004) were also found to be significant (**Figure 6B**). Conversely, using the Stepwise method, the variable immunotypes was the only factor that remained significant (*p* = 0.029) (**Figure 6B**). The proportional hazards assumption was validated for immunotypes (p > 0.05).

## 4 Discussion

The current monitoring methods for patients with STS lack effectiveness, prompting the need for alternative approaches. While previous studies have linked patient survival to the immune environment within tumor sites, our comprehension of the overall systemic immune status of STS patients remains incomplete. Utilizing peripheral blood collection as a minimally invasive means, this study aimed to assess the immune status of STS patients, providing advantages over traditional tumor sampling methods. The study aimed to evaluate immune cells, IRG, and IRSF, identify peripheral immune profiles, and investigate their association with patient survival. Consequently, the findings suggested impaired systemic immunity in STS patients, with the analysis of peripheral immunotypes indicating an impact on patient survival.

### 4.1 Contraction of B cell and CD4 T cell compartments in STS patients

Analysis of major leukocyte populations revealed significant lymphopenia accompanied by an expansion of GR, predominantly neutrophils (NEU). These observations align with findings from previous studies in STS and cancer in general (24, 30, 39–41). For example, systemic inflammation indices like the neutrophil-to-lymphocyte ratio (NLR) have been proposed as prognostic factors in STS and cancer overall (42–46). A high NLR, indicative of NEU expansion and LY reduction, is commonly associated with a poorer prognosis. The decrease in LY counts may be attributed to various factors, including the tumor’s peripheral effects (47–49) and chemotherapy-induced decline (50), which is noteworthy considering that most patients in our study were undergoing chemotherapy.

Besides the studies highlighting the significance of major leukocyte populations, there exists a gap in research investigating in-depth analyses of immune cell populations, IRG, or IRSF in the peripheral blood of STS patients. Therefore, our study uncovered compelling findings. The decrease in B cells and CD4 T cells, consistent with the observations by Kim et al. (51), along with the reduced gene expression levels of ICOSLG and CD40LG, which are positively associated with both B cells and CD4 T cells, suggests impaired activation of these cell types. B cells have been implicated in the context of STS within the tumor site, where higher infiltration and the presence of B cell-rich tertiary lymphoid structures (TLS) correlate with improved clinical outcomes (17, 18, 52). The peripheral reduction of B cells may hinder their migration to the tumor site, potentially weakening immune responses in the tumor microenvironment (TME). Decreased circulating levels of CD4 T cells have also been documented in other solid tumors (53, 54), suggesting a potential decrease in CD4 T cell infiltration, particularly Th1 cells crucial for effective immune responses. It’s worth noting that reduced levels in both populations may be associated with chemotherapy (50). However, only B cells exhibited a significant reduction compared to patients not undergoing chemotherapy (DX patients), indicating that the decrease in CD4 T cells is not solely attributable to chemotherapy. Moreover, analysis based on chemotherapy regimens revealed that ANTHRA patients exhibited the lowest frequency of B cells, consistent with a study in breast cancer demonstrating the impact of anthracyclines on the B cell compartment (55). Interestingly, TRAB patients exhibited significantly higher levels of B cells, suggesting a potential advantage of trabectedin over anthracyclines.

In addition to the decrease in B cells and CD4 T cells, the gene expression levels of ICOSLG and CD40LG were also found to be reduced in these patients. Both ICOS/ICOS-L and CD40/CD40LG play critical roles in the communication between B cells and CD4 T cells, among other immune functions (56, 57). Furthermore, the downregulation of these pathways has been implicated in cancer (58, 59). For instance, in LS, there’s evidence of a correlation between ICOS expression in tumors and improved clinical outcomes (60), which aligns with the tendency for decreased plasma levels of ICOS-L observed in our study’s PD patients. Additionally, LS patients exhibited increased frequencies of B cells. Furthermore, other immune-related genes like CD27, associated with switch memory B cells, and CD28 and IL2RA, linked to T cells, were also found to be decreased in the peripheral blood of these patients. Therefore, beyond the decrease in frequencies, the observed reduction in B cells, CD4 T cells, and the mentioned IRG suggests impaired activation of circulating B and CD4 T cells in STS patients, potentially leading to diminished migration of effector cells to the TME.

### 4.2 The major impact of immunosuppression (MDSC and Treg) in STS patients

Furthermore, an increase in suppressor populations, notably M-MDSC and memory Treg cells, was observed, along with elevated gene expression levels of the IRG ARG1 and increased plasma levels of VISTA, TIMD-4, and IL-10. In studies involving sarcoma patients, elevated levels of M-MDSC have been linked to reduced treatment efficacy, tumor growth, and a poorer prognosis (51, 61). Additionally, increased gene expression levels of ARG1 were found to be positively correlated with the heightened frequency of M-MDSC in this study. M-MDSC are known to be robust producers of arginase-1, which can inhibit NK and T cell cytotoxicity by depleting arginine from the microenvironment (62–65). Moreover, in STS, both ARG1 and ARG2 gene expression have been identified in tumor samples, suggesting an immunosuppressive TME that may impede an effective immune response (66). Hence, beyond the TME, it can be hypothesized that the expansion of M-MDSC leads to the release of arginase-1 into the peripheral microenvironment, inhibiting the cytotoxic function of T and NK cells and thereby contributing to impaired systemic immunity.

Moreover, an increase in plasma levels of the immune checkpoint VISTA was observed. This molecule has been proposed as a significant factor for immunotherapy in STS, as its expression on tumor samples has been associated with tumor grade, tumor-infiltrating lymphocyte numbers, and PD-1 expression (67). Particularly in SS, the expression of VISTA by macrophages has been shown to inhibit the infiltration of T cells in *ex vivo* experiments (68). Additionally, VISTA may influence the differentiation of MDSC (69, 70). Therefore, considering the expansion of M-MDSC, increased levels of VISTA may potentially promote the differentiation and expansion of circulating M-MDSC.

Furthermore, an increase in memory Treg cells and IL-10 plasma levels was observed. In STS and other solid tumors, the presence of Treg cells in tumor samples has been associated with worse outcomes (19, 71), suggesting a similar scenario in the periphery. Hence, the increased levels of memory Tregs, besides the reduced levels of CD4 T cells, may contribute to an immunosuppressive microenvironment. Studies have reported that M-MDSC may promote the expansion of Treg cells via the release of IL-10 into the environment (72–74), aligning with the observations for M-MDSC, memory Treg cells, and IL-10 in this study. Increased plasma levels of IL-10 have been documented in pediatric STS patients and are associated with advanced disease, poor response to chemotherapy, and unfavorable outcomes (75). IL-10 has been correlated with increased suppression of T cells in cancer patients and associated with worse survival (76, 77). Additionally, increased plasma levels of TIMD-4 were also observed in this study. TIMD-4, or TIM-4, is another immune checkpoint molecule involved in T cell regulation. In cancer, its expression in tumor samples has been correlated with worse patient outcomes due to decreased effector function of tumor-infiltrating CD8 T cells (78, 79). Although studies evaluating this molecule in STS are rare, a case report of LS showed expression of TIM-3 or TIM-4 in tumor samples, indicating a direct involvement in cancer progression (80). Considering the findings for M-MDSC, ARG1, soluble VISTA, Treg cells, IL-10, and soluble TIMD-4, it can be suggested that immunosuppression at the periphery has a significant impact, sustaining impaired systemic immunity, which may limit the anti-tumoral immune response at the tumor site.

### 4.3 Compromised cytotoxic potential associated with CD56^dim^ NK cells and CD8 T cells in STS patients

Furthermore, a decrease in CD56^dim^ NK cells and a reduction in the gene expression levels of cytotoxic-related factors PRF1, GZMB, and KLRK1 were observed. NK cells are professional killer cells crucial for tumor cell clearance, with their infiltration within tumors typically associated with better prognoses (81). NK cells can be categorized into two major subpopulations: CD56^bright^ NK cells, which are adept at secreting cytokines and chemokines, and CD56^dim^ NK cells, which exhibit greater cytotoxic activity (82). Therefore, the decreased frequency of CD56^dim^ NK cells may imply a diminished cytotoxic potential of circulating NK cells in STS patients. The observation of increased levels of CD56^bright^ NK cells and decreased levels of CD56^dim^ NK cells in PD patients further supports this assumption. Similarly, CD8 T cells are known for their cytotoxic activity. In this study, an expansion of circulating CD8 T cells was observed, suggesting an increased presence of these cells in the periphery. However, there was no observed increase in the effector CD8 T cell subpopulations EM and EMRA, indicating that despite the expansion of total CD8 T cells, there isn’t a proportional increase in the cells with the capacity to clear tumor cells. Additionally, a decrease in the gene expression levels of important cytotoxic factors such as PRF1, GZMB, and KLRK1 was noted. PRF1 was found to be positively correlated with CD56^dim^ NK cells, while PRF1, GZMB, and KLRK1 were significantly correlated with EMRA CD8 T cells. PRF1 and GZMB encode pore-forming and cytotoxic granules, respectively, involved in the cytotoxic process of NK and T cells against tumor cells (83–86). Therefore, the decreased gene expression of both may indicate an ineffective cytotoxic capacity of circulating NK and T cells. Moreover, significantly higher levels of PRF1 were observed in TRAB patients compared to ANTHRA patients. Trabectedin has demonstrated immunomodulatory effects by inhibiting tumor-associated macrophages and inducing NK-mediated cytotoxicity in cancer, as multiple myeloma (87–89). This suggests that trabectedin may enhance NK and T cell function by mitigating the effects of the TME on the systemic immune response. This could also contribute to some of the advantages of trabectedin over anthracyclines in STS treatment.

Indeed, a previous study demonstrated that peripheral NK cells from STS patients are dysfunctional, as they are unable to lyse tumor cells *in vitro* (90). Lower frequencies of circulating CD8 T cells producing PRF1 were observed in gastric cancer compared to healthy individuals (91). Additionally, lower gene expression levels of KLRK1, which encodes the activatory receptor NKG2D expressed by both NK and T cells, were noted. This aligns with previous reports showing decreased expression of NKG2D by circulating NK cells and the association of NKG2D+ CD8 T cells with improved disease-free survival in STS patients (90). Moreover, *in vitro* studies have shown that NKG2D mediates NK cell cytotoxic activity against sarcoma cells (92). Interestingly, a negative correlation was observed between the gene expression levels of PRF1, GZMB, and KLRK1 and the plasma levels of arginase-1. Considering the role of arginase-1, possibly released by M-MDSC, in inhibiting the cytotoxic capacity of NK and T cells (3, 62–65), this finding further supports the proposed immunosuppressed systemic immunity sustained by the expansion of M-MDSC. This expansion leads to the inhibition of NK and T cell cytotoxicity via the release of arginase-1 and the depletion of arginine from the circulation. Taken together, these findings suggest a decreased cytotoxic capacity of NK and T cells in the peripheral blood of STS patients, likely influenced by pro-tumoral mediators in circulation.

### 4.4 Immunotype classification and impact on patient survival

Next, it was employed unsupervised clustering analysis using immune cells, IRG and IRSF, and three distinct peripheral immune profiles (P1, P2, and P3), based on immune-related factors (C1 and C2), were identified in this cohort of STS patients. The contribution of each immune-related factor delineate the differences in the immune profiles among patients, and the investigation into the factors comprising each cluster (C1 and C2) unveiled unique associations. Cluster C1 showed associations of GR, PMN-MDSC, Th2 cells, and *naïve* CD8 T cells, with IRG such as ARG1, and IRSF like soluble IL-10, VISTA, and TIMD-4, alongside other immune checkpoint molecules and inflammatory mediators. GO pathway analysis suggested a potential association with immune suppression, particularly with Treg cells. In contrast, cluster C2 displayed associations between MO, DC, LY, T cells, EM CD4 T cells, and Th1 cells with IRG like GZMB and CD40LG, as well as the soluble factor ICOS-L, among others, correlated with cytotoxicity. GO pathway enrichment indicated a correlation with cytotoxicity, particularly associated with NK cells. These findings highlight distinct immune profiles in STS patients, providing insights into potential mechanisms underlying immune responses and suggesting avenues for patient classification and immune monitoring.

Although circulating Treg cells did not show a correlation with patient survival in this study, the observed expansion of memory Tregs may be associated with these findings, contributing to heightened immunosuppression of systemic immunity in STS, aligning with studies in STS showing that Treg cell infiltration in tumors reflects an increased risk of local recurrence (19). Additionally, the correlation patterns between GR and IRSF underscore the importance of GR in sustaining an inflammatory microenvironment in STS, aligning with findings from other studies in the field (43, 44, 93). On the other hand, both NK cells and CD8 T cells are renowned for their potent anti-tumor activity and have been extensively investigated for their ability to eliminate tumor cells (94, 95). In the context of STS, the presence of infiltrating NK cells and CD8 T cells has been linked to increased survival (96, 97). While the involvement of the ICOS-L pathway in T cells has been previously explored, it is crucial to highlight the protective nature of Th1 cells and DC, both of which exhibit a significant positive correlation with ICOS-L in this study. This correlation suggests that, despite limited studies in the context of STS, the heightened activation of Th1 cells by DC through the ICOS-L pathway might also play a crucial role in disease management and control.

Therefore, based on the immune factors in clusters C1 and C2, patients were categorized into “immune high,” “immune intermediate,” and “immune low” immunotypes. “Immune high” patients (P1) showed elevated cytotoxic-associated factors and lower inflammatory or immunosuppression-related factors, while “immune low” patients (P3) exhibited the opposite pattern. P2 patients fell into the “immune intermediate” category. Analysis of survival rates revealed that “immune high” patients had a significantly better survival outcome compared to “immune low” patients, with a 12-month survival rate of 100% versus 60%, respectively. “Immune intermediate” patients showed survival rates in-between the other two groups. This highlights the potential of peripheral immunotypes as biomarkers for predicting outcomes in STS.

Patients classified as “immune high” exhibited elevated levels of effector memory (EM) CD4 T cells, along with increased expression of CD40LG and ICOS-L. These markers suggest that B cells and CD4 T cells in these patients maintain robust functionality, which is crucial for effective immune responses. Additionally, these patients showed heightened levels of cytotoxic activity markers like GZMB, indicating a more vigorous and effective immune attack against tumor cells. In contrast, patients categorized as “immune low” displayed higher levels of suppressive factors commonly associated with Treg cells and MDSCs, such as the IRG ARG1 and plasma cytokine IL-10. The presence of these suppressive factors implies a compromised immune response, likely due to immune suppression mechanisms that inhibit effective anti-tumor activity. These observations highlight the critical role of immune mechanisms in influencing patient survival, with “immune high” subtypes benefiting from a more active and functional immune system, whereas “immune low” subtypes face challenges from immune suppression.

Recent years have seen a growing interest in incorporating diverse immune-related parameters to explore their predictive value in cancer survival and therapy response (98). In sarcoma patients, tumoral immunotypes have been proposed to optimize therapeutic strategies (27). For instance, in US, unsupervised clustering analysis of tumor samples identified three distinct immunological clusters also labeled as “immune high,” “immune intermediate,” and “immune low” (99). These clusters showed significant associations with overall survival in primary tumors. Moreover, comprehensive immune profiling has revealed LMS with an active and “hot” TME, highlighting the importance of immune competence for an effective anti-tumoral response (100). Beyond the analysis of tumor samples, peripheral immune profiles have also shown correlations with patient survival in various cancers (101–103), and in STS, gene expression profiles from TCGA databases have identified immune signatures linked to clinical outcomes (104). Additionally, in patients with US, an “immune-high” profile has been linked to a favorable response to ICI therapy (105).

The multivariate analysis identified CD40LG gene expression and PMN-MDSC frequency as significant predictors of patient survival. Although the overall model incorporating all variables did not significantly predict patient survival, the identification of these specific markers (CD40LG and PMN-MDSC) underscores their potential clinical relevance. Moreover, when discussing the advantages of utilizing peripheral phenotypes over individual-related parameters, it is important to emphasize the robustness of immunotypes in multivariate analyses. In this study, immunotypes retained their significance even after adjusting for common clinical and personal characteristics, demonstrating their ability to capture relevant information that might be overlooked when only individual variables are considered. Specifically, when using Cox regression models to evaluate individual immune cells, IRG, and IRSF, peripheral immunotypes provide a distinct advantage. They integrate a broader spectrum of immune-related data, thereby offering a more comprehensive and stable measure of immune status compared to isolated individual parameters.

Thus, the incorporation of immune profiles into prognostic models may improve patient outcomes, treatment regimens, and risk stratification. Immune profiling is pivotal for identifying patients with heightened immune cytotoxicity who may benefit from immunotherapeutic interventions to boost anti-tumor immune responses. Conversely, those with low immune activity and higher immunosuppression may need strategies to overcome immune evasion and restore function, enabling more precise patient management and tailored therapies based on their immunotype. While histological classification might have a reduced impact on observed patient immunotypes, recent studies underscore significant immune-related differences among STS histotypes (105–109), potentially correlating with varying sensitivity to immune responses and tumor aggressiveness. For instance, investigations into ICI therapy in STS patients reveal promising treatment responses in specific histotypes such as US and LMS. The independence of immunotypes from histological classification presents a significant advantage for monitoring STS patients, allowing for patient-specific categorization within the disease’s inherent heterogeneity. Yet, specific alterations in immune parameters were observed, suggesting that monitoring these changes could also complement histotype classification. Moreover, the prevalence of the “immune low” immunotype is higher in PD patients, aligning with lower survival rates. Despite the suggested impact of trabectedin in promoting improved systemic immunity, no discernible effect was noted for immunotype classification. This is crucial for therapeutic interventions, where shifts in immune-related factors might correlate directly with treatment responses.

In conclusion, this study revealed contraction and impairment of circulating B and CD4 T cells, expansion of suppressor cells such as M-MDSC and Treg, and increased levels of immune-related factors associated with inhibition, including ARG1, soluble VISTA, soluble TIMD-4, and IL-10. Moreover, compromised cytotoxic function was observed due to reductions in cytotoxic factors like PRF1 and GZMB, along with cytotoxic NK cells and activatory receptors such as KLRK1 (NKG2D), indicating compromised systemic immunity in STS patients. Unsupervised clustering analysis identified three distinct immunotypes, each characterized by varying levels of immunosuppression or activation and cytotoxicity-related factors. Patients (P1) with lower levels of immunosuppressor factors (C1) and higher levels of factors related to the activation and cytotoxicity of NK and T cells (C2) exhibited superior survival rates compared to patients (P3) with the opposite pattern. These findings suggest impaired immunity in STS patients with impact on patient survival, highlighting the potential of monitoring STS patients using peripheral blood samples to evaluate the immune status of a patient as an alternative to tumor sample evaluation. Additionally, classifying STS patients into more homogeneous groups may streamline clinical management.

This study provides valuable insights into the peripheral immune landscape in STS patients, but several limitations must be acknowledged. The small sample size limits the statistical power and the ability to accurately evaluate clinical parameters, emphasizing the need for larger cohorts. Additionally, the variability in diagnostic timing and non-standardized sample collection times introduce heterogeneity, making it challenging to isolate newly diagnosed patients and impacting the consistency of the data. The cross-sectional design further restricts the study by not capturing immune dynamics over time, which is crucial for understanding disease progression and treatment response. Without external validation of the immunotypes identified, the generalizability of these findings remains uncertain, suggesting a need for future studies to validate these results in larger, diverse cohorts.

Building on the current findings, future research should focus on longitudinal studies to monitor changes in the immune landscape over time and under different treatment regimens. This approach will provide deeper insights into the prognostic and predictive significance of immune profiling in STS. Additionally, there is a need for interventions aimed at modulating peripheral immune responses, which could potentially improve clinical outcomes. Future trials should aim to standardize and validate immunotype classification, ultimately integrating these insights into clinical practice to enhance the management of STS patients.

## Data Availability

The original contributions presented in the study are included in the article/**Supplementary Materials**. Further inquiries can be directed to the corresponding author.
