# Supplementary material for: Peripheral immune profiling of soft tissue sarcoma: perspectives for disease monitoring
Source: Front Immunol. 2024 Oct 21;15:1391840. doi: 10.3389/fimmu.2024.1391840 (PMC11536262; doi:10.3389/fimmu.2024.1391840)
Supplement: Supplementary file 1 [file DataSheet1.pdf]

Supplementary material

Supplementary file S1. Detailed demographic and clinical characteristics of STS patients enrolled in the study.

Sample ID	Sex	Age	Metastatic disease	Recurrence	General localization	Specific localization	Histological classification	Treatment/response	Therapy	Status	TAD (months)	TAC (months)
1	M	64	YES	YES	Extremity	Lower limb	Liposarcoma	Progression disease	Anthracycline-based therapy followed by trabectedin-based therapy	DOD	49	5
2	M	62	YES	YES	Trunk	Retroperitoneum	Liposarcoma	Progression disease	Other	DOD	49	11
3	F	21	YES	YES	Extremity	Lower limb	Clear cell sarcoma	Progression disease	Anthracycline-based therapy	DOD	21	0
4	F	46	NO	YES	Trunk	Trunk	Undifferentiated sarcoma	Progression disease	Other	DOD	29	1
5	F	56	YES	NO	Trunk	Pelvis	Leiomyosarcoma	Progression disease	Other	DOD	22	0
6	F	53	YES	YES	Gynecologic tumors	Uterus	Leiomyosarcoma	Progression disease	Anthracycline-based therapy followed by trabectedin-based therapy	DOD	60	13
7	M	77	YES	NO	Trunk	Adrenal gland	Haemangiosarcoma	Progression disease	Anthracycline-based therapy followed by trabectedin-based therapy	DOD	23	0
8	M	19	YES	YES	Head and Neck	Head and Neck	Synovial sarcoma	Progression disease	Trabectedin-based therapy	DOD	6	1
9	F	68	YES	NO	Trunk	Pelvis	Leiomyosarcoma	Stable disease	Trabectedin-based therapy	AWD	101	50
10	M	65	NO	NO	Trunk	Retroperitoneum	Leiomyosarcoma	Progression disease	Other	DOD	34	18
11	M	34	NO	NO	Trunk	Trunk	Alveolar soft part sarcoma	Stable disease	Other	AWD	63	48
12	F	75	NO	YES	Extremity	Lower limb	Liposarcoma	Stable disease	Trabectedin-based therapy	DOC	60	9
13	F	78	YES	NO	Gynecologic tumors	Uterus	Leiomyosarcoma	Stable disease	Trabectedin-based therapy	DOD	47	29
14	M	57	YES	NO	Trunk	Jejunum	Leiomyosarcoma	Progression disease	Anthracycline-based therapy followed by trabectedin-based therapy	DOD	31	16
15	F	43	YES	NO	Gynecologic tumors	Uterus	Leiomyosarcoma	Progression disease	Other	DOD	5	3
16	M	28	YES	NO	Extremity	Upper limb	Leiomyosarcoma	Progression disease	Anthracycline-based therapy followed by trabectedin-based therapy	DOD	46	1
17	F	33	YES	YES	Extremity	Lower limb	Rhabdomyosarcoma	Progression disease	Anthracycline-based therapy	DOD	6	1
18	F	62	NO	YES	Trunk	Retroperitoneum	Leiomyosarcoma	Stable disease	Anthracycline-based therapy followed by trabectedin-based therapy	AWD	92	45
19	M	75	YES	NO	Extremity	Upper limb	Liposarcoma	Stable disease	Anthracycline-based therapy followed by trabectedin-based therapy	DOC	72	12
20	M	62	YES	YES	Extremity	Lower limb	MPNET	Progression disease	Other	DOD	108	27
21	M	50	YES	NO	Extremity	Lower limb	Synovial sarcoma	Progression disease	Anthracycline-based therapy followed by trabectedin-based therapy	DOC	23	1
22	M	57	NO	NO	Trunk	Retroperitoneum	Leiomyosarcoma	Stable disease	Trabectedin-based therapy	AWD	47	28
23	F	58	NO	NO	Extremity	Lower limb	Undifferentiated sarcoma	Surgery	NA	DOD	28	1
24	F	53	YES	NO	Gynecologic tumors	Uterus	Leiomyosarcoma	Surgery	NA	AWD	28	28
25	F	59	YES	NO	Gynecologic tumors	Uterus	Leiomyosarcoma	Stable disease	Anthracycline-based therapy followed by trabectedin-based therapy	DOD	74	14
26	F	46	YES	NO	Gynecologic tumors	Uterus	Leiomyosarcoma	Progression disease	Anthracycline-based therapy followed by trabectedin-based therapy	AWD	63	28
27	M	52	YES	YES	Extremity	Lower limb	Synovial sarcoma	Progression disease	Anthracycline-based therapy followed by trabectedin-based therapy	DOD	38	9
28	M	75	YES	YES	Trunk	Thorax	Undifferentiated sarcoma	Stable disease	Anthracycline-based therapy followed by trabectedin-based therapy	AWD	42	28
29	F	59	NO	NO	Trunk	Thorax	Haemangiosarcoma	Stable disease	Anthracycline-based therapy	AWD	35	28
30	M	63	YES	YES	Extremity	Lower limb	MPNET	Progression disease	Other	DOD	108	13
31	F	50	YES	NO	Trunk	Retroperitoneum	Leiomyosarcoma	Progression disease	Anthracycline-based therapy followed by trabectedin-based therapy	AWD	44	28
32	M	54	YES	NO	Extremity	Lower limb	Undifferentiated sarcoma	Progression disease	Anthracycline-based therapy followed by trabectedin-based therapy	AWD	144	28
33	F	76	YES	NO	Trunk	Retroperitoneum	Leiomyosarcoma	Stable disease	Trabectedin-based therapy	DOC	18	3
34	M	36	NO	NO	Trunk	Trunk	Clear cell sarcoma	Progression disease	Other	AWD	33	21
35	M	29	YES	NO	Trunk	Thorax	MPNET	Stable disease	Anthracycline-based therapy followed by trabectedin-based therapy	AWD	89	20
36	M	79	NO	NO	Trunk	Pelvis	Undifferentiated sarcoma	Stable disease	Trabectedin-based therapy	AWD	24	20
37	F	56	YES	NO	Trunk	Heart	Liposarcoma	Stable disease	Anthracycline-based therapy followed by trabectedin-based therapy	AWD	28	20
38	F	43	YES	NO	Trunk	Retroperitoneum	Leiomyosarcoma	Stable disease	Trabectedin-based therapy	AWD	60	20
39	F	49	YES	NO	Extremity	Lower limb	Synovial sarcoma	Stable disease	Anthracycline-based therapy followed by trabectedin-based therapy	AWD	24	11
40	M	52	NO	NO	Gynecologic tumors	Spermatic cord	Liposarcoma	Stable disease	Anthracycline-based therapy followed by trabectedin-based therapy	AWD	24	11
41	M	54	YES	NO	Extremity	Lower limb	Synovial sarcoma	Stable disease	Anthracycline-based therapy followed by trabectedin-based therapy	AWD	42	11
42	F	13	YES	NO	Extremity	Lower limb	Synovial sarcoma	Stable disease	Anthracycline-based therapy	AWD	161	11
43	M	66	NO	NO	Extremity	Lower limb	Undifferentiated sarcoma	Stable disease	Anthracycline-based therapy	AWD	19	9
44	F	44	YES	NO	Trunk	Liver	Embryonal sarcoma	Stable disease	Anthracycline-based therapy	AWD	15	9
45	F	52	YES	NO	Gynecologic tumors	Uterus	Leiomyosarcoma	Stable disease	Other	AWD	18	9
46	F	58	NO	NO	Trunk	Thorax	Leiomyosarcoma	Stable disease	Other	AWD	13	9
47	F	54	YES	NO	Gynecologic tumors	Uterus	Endometrial stromal sarcoma	Stable disease	Anthracycline-based therapy	AWD	13	9
48	M	46	NO	NO	Extremity	Lower limb	Synovial sarcoma	Stable disease	Anthracycline-based therapy	AWD	18	9
49	F	60	YES	NO	Trunk	Thorax	Liposarcoma	Surgery	NA	DOD	11	11
50	M	56	NO	NO	Extremity	Lower limb	Undifferentiated sarcoma	Stable disease	Anthracycline-based therapy	AWD	27	22
51	F	61	NO	NO	Trunk	Pelvis	Undifferentiated sarcoma	Surgery	NA	AWD	20	21
52	F	52	NO	NO	Extremity	Lower limb	Undifferentiated sarcoma	Surgery	NA	AWD	17	17
53	M	82	NO	YES	Extremity	Lower limb	Liposarcoma	Surgery	NA	AWD	16	16
54	F	54	NO	NO	Extremity	Lower limb	Liposarcoma	Surgery (	NA	AWD	11	11
55	M	60	NO	NO	Extremity	Lower limb	Synovial sarcoma	Surgery	NA	AWD	5	5

Legend: MPNET - malignant peripheral nerve sheath tumor; AWD – alive with disease; DOD – death of disease; DOC – death of other causes; TAD – time after diagnosis (primary tumor); TAC – time after sample collection; NA – not applicable

Supplementary file S2. Monoclonal antibodies used to perform multiparametric flow cytometry analysis.

Antibody	Conjugate	Clone	Brand	Cat#	RRID
**CD3**	V450	ICHT1	BD Horizon™	561416	AB_10612021
**CD3**	PerCp-Cy5.5	HIT3a	BioLegend®	300328	AB_1575008
**CD4**	PerCp-Cy5.5	OKT4	BioLegend®	317428	AB_1186124
**CD8**	APC-H7	HIT8a	BD Biosciences™	641400	AB_1645736
**CD11b**	APC	ICRF44	BioLegend®	301310	AB_2564134
**CD11c**	PE-Cy7	B-ly6	BD Pharmingen™	561356	AB_10611859
**CD14**	V450	MOP9	BD Horizon™	560349	AB_1645559
**CD15**	PE-Cy7	HI98	BD Pharmingen™	560827	AB_10563901
**CD16**	APC-Cy7	3G8	BioLegend®	302018	AB_314218
**CD19**	APC-H7	SJ2501	BD Pharmingen™	560177	AB_1645470
**CD19**	PerCp-Cy5.5	HIB19	BioLegend®	302230	AB_2275547
**CD20**	APC-H7	2H7	BD Pharmingen™	560734	AB_1727449
**CD24**	FITC	ML5	BD Pharmingen™	555427	AB_395821
**CD25**	PE	M-A251	BD Pharmingen™	555432	AB_395826
**CD27**	PE-Cy7	M-T271	BD Pharmingen™	560609	AB_1727456
**CD33**	PE	WM53	BD Pharmingen™	555450	AB_395843
**CD38**	APC	HIT2	BD Pharmingen™	555462	AB_398599
**CD45**	FITC	HI30	BD Pharmingen™	555482	AB_395874
**CD45RA**	PE-Cy7	5H9	BD Pharmingen™	561216	AB_10611721
**CD45RO**	APC-H7	UCHL1	BD Pharmingen™	561137	AB_10562194
**CD56**	PerCp-Cy5.5	HCD56	BD Pharmingen™	560842	AB_2033964
**CD56**	PE	B159	BioLegend®	318306	AB_604101
**CD123**	PerCp-Cy5.5	6H6	BioLegend®	306016	AB_2264693
**CD127**	AF647	HIL-7R-M21	BD Pharmingen™	558598	AB_647113
**CD183 (CXCR3)**	PE	IC6/CXCR3	BD Pharmingen™	550633	AB_2292853
**CD194 (CCR4)**	PE-Cy7	IG1	BD Biosciences™	557864	AB_396907
**CD196 (CCR6)**	PE-Cy7	11A9	BD Pharmingen™	560620	AB_1727440
**CD197 (CCR7)**	PE	150503	BD Pharmingen™	560765	AB_2033949
**CD274 (PD-L1)**	FITC	MIH1	BD Pharmingen™	558065	AB_647176
**CD279 (PD-1)**	FITC	MIH4	BD Pharmingen™	557860	AB_2159176
**CD279 (PD-1)**	APC	MIH4	BD Pharmingen™	558694	AB_1645458
**CD279 (PD-1)**	PE	MIH4	BD Pharmingen™	557946	AB_647199
**HLA-DR**	V500	G46-6	BD Horizon™	561224	AB_10563765
**IgD**	V500	IAS6-2	BD Horizon™	561490	AB_10679356

Legend: RRID - research resource identification

Supplementary file S3. Gating strategy used for multiparametric flow cytometry data analysis. A) Gating strategy for T cells, B cells, NK cells and DC, including subpopulations. [1a – 1c] Debris exclusion and leucocytes (LEU) identification; lymphocytes (LY) selection; doublets exclusion. [2a] T cells identification. [2b] Identification of double negative (DN), double positive (DP, CD4 and CD8 T cells. [2c 1] Identification of CD4 T cells subpopulations, naïve (CD45RA+ CCR7+), CM (CD45- CCR7+), EM (CD45RA- CCR7-), and EMRA (CD45RA+ CCR7-) CD4 T populations. [2c 2] Identification of CD8 T cells subpopulations, naïve (CD45RA+ CCR7+), naïve low ($CD45RA^{low}$ CCR7+), CM (CD45- CCR7+), EM (CD45RA- CCR7-), EMRA low ($CD45RA^{low}$ CCR7-), and EMRA (CD45RA+ CCR7-). [3a] Identification of Th1 (CXCR3+ CCR6-), Th17 (CX3CR1- CCR6+) and Th2 cells (CX3CR1- CCR6-). [3b – 3c] Identification of HLA-DR+ cells and PD-1+ cells. [4a – 4c] Identification of regulatory T (Treg) cells. [4d] Identification of naïve and memory Treg cells. [5a] Identification of B cells. [5b] Identification of B cells subpopulations, naïve (IgD+ CD27-), pre-SM (IgD+ CD27+), SM (IgD- CD27+) and exh (IgD- CD27-) cells. [5c] Plasmablast identification. [5d] Transitional B cells identification. [6a – 6c] Identification of NK cells and NKT-like cells. [7a – 7d] Identification of dendritic cells (DC). [7e] Identification of DC subpopulations, myeloid DC (mDC) and plasmacytoid DC (pDC). [7f] Identification of mDC subpopulations, mDC type I (mDC I), and mDC type II (mDC II). B) Gating strategy for myeloid-derived suppressor cells (MDSC). [1 – 5] Identification of MDSC. [6] Identification of MDSC subpopulations, early-MDSC (e-MDSC, CD14-, CD15-), monocytic-MDSC (M-MDSC, CD14+, CD15-/+) and polymorphonuclear-MDSC (PMN-MDSC, CD14-, CD15+).

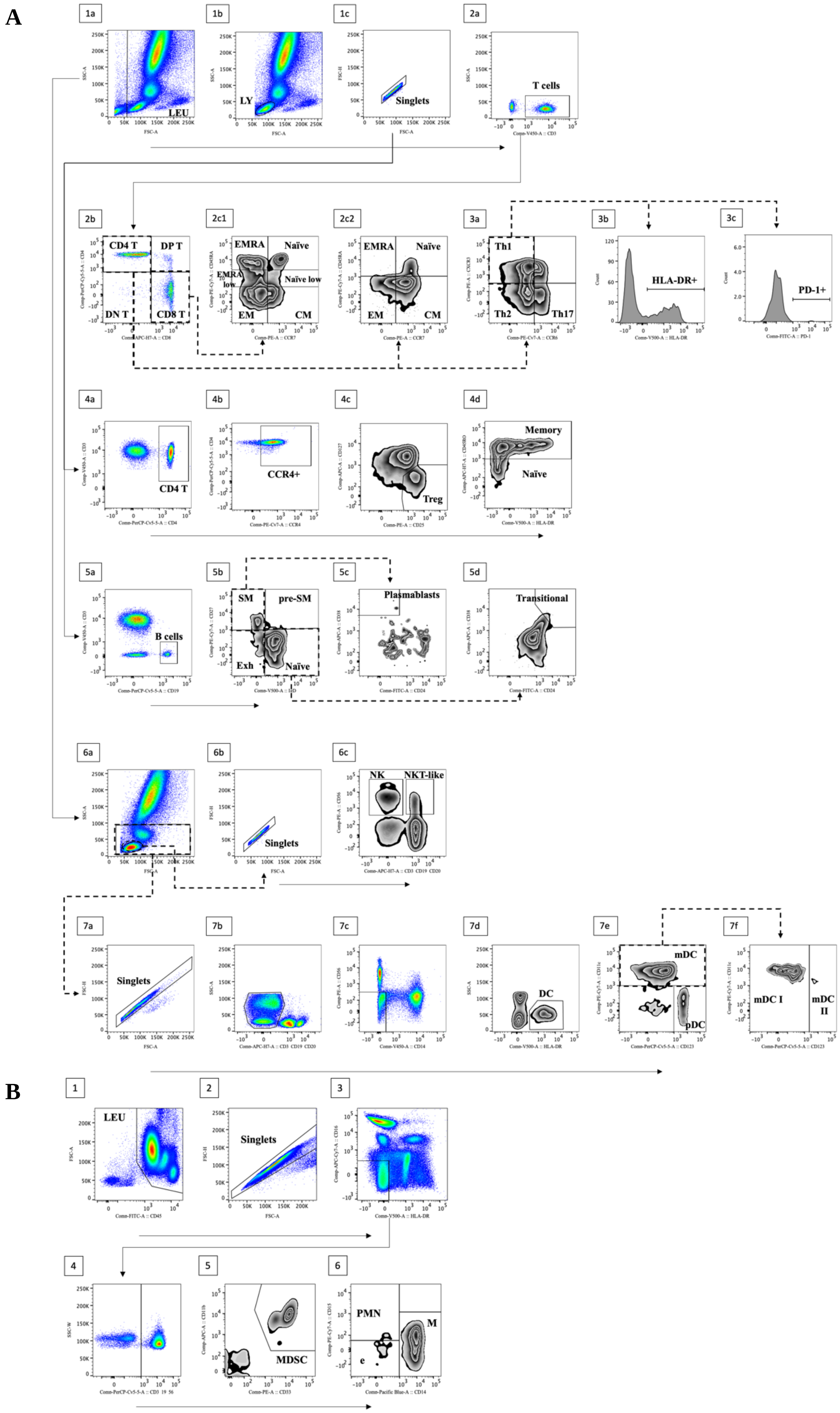
A
1a
LEU
1b
LY
1c
Singlets
2a
T cells
2b
CD4 T
DP T
DN T
CD8 T
2c1
EMRA
Naïve
EMRA low
Naïve low
EM
CM
2c2
EMRA
Naïve
EM
CM
3a
Th1
Th2
Th17
3b
HLA-DR+
3c
PD-1+
4a
CD4 T
4b
CCR4+
4c
Treg
4d
Memory
Naïve
5a
B cells
5b
SM
pre-SM
Exh
Naïve
5c
Plasmablasts
5d
Transitional
6a
6b
Singlets
6c
NK
NKT-like
7a
Singlets
7b
7c
7d
DC
7e
mDC
pDC
7f
mDC I
mDC II
B
1
LEU
2
Singlets
3
4
5
MDSC
6
PMN
M
e

Supplementary file S4. Primers used to preform gene expression analysis of immune-related genes (IRG) by real-time reverse transcription-quantitative polymerase chain reaction (RT-qPCR).

Gene name	NCBI Gene id	GenBank Accession nº	Primer Forward	Primer Reverse	PrimerBank ID or reference
REFERENCE GENES					
**ACTB**	60	NM_001101	CTGGAACGGTGAAGGTGACA	AAGGGACTTCCTGTAACAATGCA	4501885a1 (1)
**B2M**	567	NM_004048	TGCTGTCTCCATGTTTGATGTATCT	TCTCTGCTCCCCACCTCTAAGT	37704380c1 (1)
**RPL13A**	23521	NM_012423	CCTGGAGGAGAAGAGGAAAGAGA	TTGAGGACCTCTGTGTATTTGTCAA	14591905c1 (1)
GENES OF INTEREST					
**ARG1**	383	NM_000045	GTGGAAACTTGCATGGACAAC	AATCCTGGCACATCGGGAATC	346986433c1
**B3GAT1**	27087	NM_018644	CCTGGCGTGGTCTACTTCG	GCAGGTTGACGGCAAATCC	77695913c1
**BTLA**	151888	NM_001085357	CATCTTAGCAGGAGATCCCTTTG	GACCCATTGTCATTAGGAAGCA	145580618c1
**BTRC**	8945	NM_003939	CCAGACTCTGCTTAAACCAAGAA	GGGCACAATCATACTGGAAGTG	379030597c1
**CCL2**	6347	NM_002982	CAGCCAGATGCAATCAATGCC	TGGAATCCTGAACCCACTTCT	4506841a1
**CCL3**	6348	NM_002983	AGTTCTCTGCATCACTTGCTG	CGGCTTCGCTTGGTTAGGAA	4506843a1
**CCL4**	6351	NM_002984	CTGTGCTGATCCCAGTGAATC	TCAGTTCAGTTCCAGGTCATACA	4506845a1
**CCL7**	6354	---	GACAAGAAAACCCAAACTCCAAAG	TCAAAACCCACCAAAATCCA	(2)
**CCL8**	6355	NM_005623	TGGAGAGCTACACAAGAATCACC	TGGTCCAGATGCTTCATGGAA	22538815c1
**CCL11**	6356	NM_002986	CCCCTTCAGCGACTAGAGAG	TCTTGGGGTCGGCACAGAT	22538399c1
**CCL22**	6367	NM_002990	ATCGCCTACAGACTGCACTC	GACGGTAACGGACGTAATCAC	300360575c1
**CCL24**	6369	NM_002991	ACATCATCCCTACGGGCTCT	CTTGGGGTCGCCACAGAAC	22165426c1
**CD27**	939	NM_001242	CAGAGAGGCACTACTGGGCT	CGGTATGCAAGGATCACACTG	117422442c1
**CD28**	940	NM_001243078	CTATTTCCCGGACCTTCTAAGCC	GCGGGGAGTCATGTTCATGTA	340545509c1
**CD226**	10666	NM_006566	GTGGAGTGGTTCAAGATCGGG	GCTTCCTTATGACCATGCCAT	171460996c1
**CD274**	29126	NM_014143	TGGCATTTGCTGAACGCATTT	TGCAGCCAGGTCTAATTGTTTT	292658763c1
**CD3D**	915	NM_001040651	ACTGGCTACCCTTCTCTCG	CCGTTCCCTCTACCCATGTGA	98985800c1
**CD3E**	916	NM_000733	CCTCTTATCAGTTGGCGTTTGG	TTCAGTGACAGGTGATCCTCA	166362733c1
**CD3G**	917	NM_000073	TGGCCCAGTCAATCAAAGGAA	CAAGTCAGAAGTACCGAACCATC	166362738c1
**CD40LG**	959	NM_000074	ACATACAACCAAACTTCTCCCCG	GCAAAAAGTGCTGACCCAATCA	58331233c1
**CD47**	961	NM_001777	AGAAGGTGAAACGATCATCGAGC	CTCATCCATACCACCGGATCT	68223312c1
**CD48**	962	NM_001778	AGGTTGGGATTCGTGTCTGG	AGTTGTTTGTAGTTCTCAGGCAG	365733591c1
**CD69**	969	NM_001781	ATTGTCCAGGCCAATACACATT	CCTCTCTACCTGCGTATCGTTTT	221554485c1
**CD96**	10225	NM_198196	CAAACACAGACAGTAGGCTTCTT	GGGGATGATAGACAGCAATCAG	93141044c1
**CDH1**	999	NM_004360	CGAGAGCTACACGTTCACGG	GGGTGTCGAGGGAAAAATAGG	169790842c1
**CSF1**	1435	NM_172210	TGGCGAGCAGGAGTATCAC	AGGTCTCCATCTGACTGTCAAT	166235149c1
**CSF2**	1437	NM_000758	TCCTGAACCTGAGTAGAGACAC	TGCTGCTTGTAGTGGCTGG	371502128c1
**CSF3**	1440	NM_001178147	GCTGCTTGAGCCAACTCCATA	GAACGCGGTACGACACCTC	296011056c1
**CTLA4**	1493	NM_005214	GCCCTGCACTCTCCTGTTTTT	GGTTGCCGCACAGACTTCA	339276048c1
**CX3CL1**	6376	NM_002996	ACCACGGTGTGACGAAATG	TGTTGATAGTGGATGAGCAAAGC	54111253c1
**CXCL1**	2919	---	GCGCCCAAACCGAAGTCATA	ATGGGGGATGCAGGATTGAG	(3)
**CXCL5**	6374	NM_002994	AGCTGCGTTGCGTTTGTTTAC	TGGCGAACACTTGCAGATTAC	41872613c1
**CXCL9**	4283	NM_002416	CCAGTAGTGAGAAAGGGTCGC	AGGGCTTGGGGCAAATTGTT	4505186c1
**CXCL10**	3627	NM_001565	GTGGCATTCAAGGAGTACCTC	TGATGGCCTTCGATTCTGGATT	323422857c1
**CXCL11**	6373	NM_005409	GACGCTGTCTTTGCATAGGC	GGATTTAGGCATCGTTGTCCTTT	307611978c1
**CXCL13**	10563	NM_006419	GCTTGAGGTGTAGATGTGTCC	CCCACGGGGCAAGATTTGAA	194733765c1
**FCGR3A**	2214	NM_000569	CCTCCTGTCTAGTCGGTTTGG	TCGAGCACCCTGTACCATTGA	24429586a1
**FGF2**	2247	NM_002006	AGAAGAGCGACCCTCACATCA	CGGTTAGCACACACTCCTTTG	153285460c1
**GZMB**	3002	NM_004131	CCCTGGGAAAACACTCACACA	GCACAACTCAATGGTACTGTCG	221625527c1
**HAVCR2**	84868	NM_032782	CTGCTGCTACTACTTACAAGGTC	GCAGGGCAGATAGGCATTCT	354681988c1
**HGF**	3082	NM_001010931	GCTATCGGGGTAAAGACCTACA	CGTAGCGTACCTCTGGATTGC	58533162c1
**HLA-DRB1**	3123	AJ297586	GAGCAGGTTAAACATGAGTGTCA	CTCTCCACAACCCCGTAGT	15387629a1
**ICOSLG**	23308	NM_015259	GCAGCCTTCGAGCTGATACTC	GTTTTCGACTCACTGGTTTGC	58331247c1
**IDO1**	3620	NM_002164	GCCAGCTTCGAGAAAGAGTTG	ATCCCAGAACTAGACGTGCAA	323668304c1
**IFNA1**	3439	NM_024013	GCCTCGCCCTTTGCTTTACT	CTGTGGGTCTCAGGGAGATCA	13128950a1
**IFNG**	3458	NM_000619	TCGGTAACTGACTTGAATGTCCA	TCGCTTCCCTGTTTTAGCTGC	56786137c1
**IL1B**	3553	NM_000576	ATGATGGCTTATTACAGTGGCAA	GTCGGAGATTCGTAGCTGGA	27894305c1
**IL2**	3558	M22005	TACAAGAACCCGAAACTGACTCG	ACATGAAGGTAGTCTCACTGCC	386818a1
**IL2RA**	3559	NM_000417	GTGGGGACTGCTCACGTTC	CCCGCTTTTTATTCTGCGGAA	269973860c1
**IL4**	3565	NM_000589	CCAACTGCTTCCCCCTCTG	TCTGTTACGGTCAACTCGGTG	4504669a1
**IL5**	3567	NM_000879	TGGAGCTGCCTACGTGTATG	TTCGATGAGTAGAAAGCAGTGC	28559032c1

**IL6**	3569	NM_000600	ACTCACCTCTTCAGAACGAATTG	CCATCTTTGGAAGGTTCAGGTTG	224831235c1
**IL7**	3574	NM_000880	TTGGACTTCCTCCCCTGATCC	TCGATGCTGACCATTAGAACAC	4504677a1
**IL8**	3576	NM_000584	TTTTGCCAAGGAGTGCTAAAGA	AACCCTCTGCACCCAGTTTTC	10834978a1
**IL9**	3578	NM_000590	CTCTGTTTGGGCATTCCCTCT	GGGTATCTTGTTTGCATGGTGG	10834980a1
**IL10**	3586	NM_000572	GACTTTAAGGGTTACCTGGGTTG	TCACATGCGCCTTGATGTCTG	24430216c1
**IL12A**	3592	NM_000882	CCTTGCACTTCTGAAGAGATTGA	ACAGGGCCATCATAAAAGAGGT	325974478c1
**IL13**	3596	NM_002188	CCTCATGGCGCTTTTGTTGAC	TCTGGTTCTGGGTGATGTTGA	26787977c1
**IL15**	3600	NM_172175	TTGGGAACCATAGATTTGTGCAG	GGGTGAACATCACTTTCCGTAT	26787986a1
**IL16**	3603	NM_172217	GCCGAAGACCCTTGGGTTAG	GCTGGCATTGGGCTGTAGA	289063450c1
**IL17A**	3605	NM_002190	TCCCACGAAATCCAGGATGC	GGATGTTCAGGTTGACCATCAC	27477085c1
**IL18**	3606	NM_001562	TCTTCATTGACCAAGGAAATCGG	TCCGGGGTGCATTATCTCTAC	342349317c1
**IL20**	50604	NM_018724	ATGAAAGCCTCTAGTCTTGCCT	GCCCCGTATCTCAGAAAATCC	50845426c1
**IL21**	59067	NM_021803	TAGAGACAAACTGTGAGTGGTCA	GGGCATGTTAGTCTGTGTTTCTG	365733583c1
**IL23A**	51561	NM_016584	CTCAGGGACAACAGTCAGTTC	ACAGGGCTATCAGGGAGCA	28144902c1
**IL27**	246778	NM_145659	ACCGCTTTGCGGAATCTCA	AGGTCAGGGAAACATCAGGGA	28416913a1
**IL31**	386653	NM_001014336	CACGTTGCCCGTCCGTTTA	TCTTCGAGAGGGACTGTAATTCC	62122910c1
**ITGAM**	3684	NM_001145808	GCCTTGACCTTATGTCATGGG	CCTGTGCTGTAGTCGCACT	224831238c1
**KLRC1**	3821	NM_007328	AGCTCCATTTTAGCAACTGAACA	CAACTATCGTTACCACAGAGGC	283046824c1
**KLRC2**	3822	NM_002260	GCCAGCATTTTACCTTCCTCA	ACTGCACAGTTAAGTTCAGCAT	4504883a1
**KLRD1**	3824	NM_007334	CAGGACCCAACATAGAACTCCA	GGAAATGAAGTAACAGTTGCACC	167614494c1
**KLRF1**	51348	NM_016523	TACTGGGAATATCTGGAACCGT	TTGAGCCATTCTGATTGGCAT	7705573c1
**LAG3**	3902	NM_002286	GCGGGGACTTCTCGCTATG	GGCTCTGAGAGATCCTGGGG	167614499c1
**LAMP1**	3916	NM_005561	TCTCAGTGAACTACGACACCA	AGTGTATGTCCTCTTCCAAAAGC	112380627c1
**LIF**	3976	NM_002309	CCAACGTGACGGACTTCCC	TACACGACTATGCGGTACAGC	380418322c1
**LTA**	4049	NM_000595	ATGACACCACCTGAACGTCTC	CTCTCCAGAGCAGTGAGTTCT	6806892c1
**MIF**	4282	---	GAACAACTCCACCTTCGCCT	CCGTTTATTTCTCCCCACCA	(4)
**MMP1**	4312	NM_002421	AAAATTACACGCCAGATTTGCC	GGTGTGACATTACTCCAGAGTTG	225543092c1
**NCAM1**	4684	NM_001076682	GGCATTTACAAGTGTGTGGTTAC	TTGGCGCATTCTTGAACATGA	336285437c1
**NCR1**	9437	NM_001242357	TGGACCCGAAGTGATCTCG	TCCTTGAGCAGTAAGAACATGC	334358898c1
**NCR2**	9436	NM_004828	GGCTCTCAGGCACAATCCAAG	GCTGAAGCCTCCTTACACCA	153945781c1
**NCR3**	259197	NM_001145467	CCCCTGAGATTCGTACCCTG	CTCCACTCTGCACACGTAGAT	224586864c1
**PDCD1**	5133	NM_005018	CCAGGATGGTTCTTAGACTCCC	TTTAGCACGAAGCTCTCCGAT	167857791c1
**PDCD1LG2**	80380	NM_025239	ATTGCAGCTTCACCAGATAGC	AAAGTTGCATTCCAGGGTCAC	190014604c1
**PRF1**	5551	NM_005041	GGCTGGACGTGACTCCTAAG	CTGGGTGGAGGCGTTGAAG	133908619c1
**SELL**	6402	NM_000655	ACCCAGAGGGACTTATGGAAC	GCAGAATCTTCTAGCCCTTTGC	262206314c1
**SLAMF7**	57823	NM_021181	ACCCTCATCTATATCCTTTGGCA	CACCAACGGAACCGACCAG	19923571c1
**TIGIT**	201633	NM_173799	TCTGCATCTATCACACCTACCC	CCACCACGATGACTGCTGT	256600227c1
**TNF**	7124	NM_000594	CCTCTCTCTAATCAGCCCTCTG	GAGGACCTGGGAGTAGATGAG	25952110c1
**TNFRSF1B**	7133	NM_001066	CGGGCCAACATGCAAAAGTC	CAGATGCGGTTCTGTTCCC	23312365c1
**TNFRSF8**	943	NM_001243	TCCACGGAGCACACCAATAAC	ACTGAGAGCATGACATCGCTG	325053721c1
**TNFRSF9**	3604	NM_001561	AGCTGTTACAACATAGTAGCCAC	GGACAGGGACTGCAAATCTGAT	315259099c1
**TNFSF9**	8744	NM_003811	GGCTGGAGTCTACTATGTCTTCT	ACCTCGGTGAAGGGAGTCC	68348710c1
**TNFSF10**	8743	NM_001190942	TGCGTGCTGATCGTGATCTTC	GCTCGTTGGTAAAGTACACGTA	300193031c1
**TNFSF12**	8742	NM_003809	GAGGGGAAGGCTGTCTACCT	GAACCTGGAAGAGTCCGAAGTA	23510442c1
**TNFSF13**	8741	NM_172088	CTCTGCTGACCCAACAAACAG	GGAGGTGGCGTTAATGGGAAC	23510442c1
**TNFSF13B**	10673	NM_001145645	GGGAGCAGTCACGCCTTAC	GATCGGACAGAGGGGCTTT	26051248a1
**TSLP**	85480	NM_138551	ATGTTCGCCATGAAAACTAAGGC	GCGACGCCACAATCCTTGTA	209954675c1
**VEGFA**	7422	NM_001171627	AGGGCAGAATCATCACGAAGT	AGGGTCTCGATTGGATGGCA	372466598c1

References:

1. Vandesompele J, De Preter K, Pattyn F, Poppe B, Van Roy N, De Paepe A, et al. Accurate normalization of real-time quantitative RT-PCR data by geometric averaging of multiple internal control genes. Genome Biol. (2002) 3(7):RESEARCH0034. doi: 10.1186/gb-2002-3-7-research0034

2. Wang G, Guan J, Li G, Wu F, Yang Q, Huang C, et al. Effect of ORF7 of SARS-CoV-2 on the Chemotaxis of Monocytes and Neutrophils In Vitro. Dis Markers. (2021) 2021:6803510. doi: 10.1155/2021/6803510.

3. Zhou X, Peng M, He Y, Peng J, Zhang X, Wang C, et al. CXC Chemokines as Therapeutic Targets and Prognostic Biomarkers in Skin Cutaneous Melanoma Microenvironment. Front Oncol. (2021) 11:619003. doi: 10.3389/fonc.2021.619003

4. Yao Y, Deng Q, Song W, Zhang H, Li Y, Yang Y, et al. MIF Plays a Key Role in Regulating Tissue-Specific Chondro-Osteogenic Differentiation Fate of Human Cartilage Endplate Stem Cells under Hypoxia. Stem Cell Reports. (2016) 7(2):249-62. doi: 10.1016/j.stemcr.2016.07.003

Supplementary file S5. Immune-related soluble factors (IRSF) evaluated by multiplex analyte profiling (xMAP® technology) in plasma samples.

ProcartaPlex™ Human Immune Monitoring Panel, 65plex

Analyte	Bead Number	[Std1] (pg/mL)	LLOQ (pg/mL)	ULOQ (pg/mL)
APRIL	88	452300	110	452300
BAFF	86	13000	3,17	13000
BLC	29	46400	11	46400
bNGF	55	22200	5,42	22200
CD30	84	38600	9,42	38600
CD40-Ligand	74	42400	10	42400
ENA-78 (LIX)	82	37500	9,16	37500
Eotaxin	33	6150	1,5	6150
Eotaxin-2	30	15000	15	15000
Eotaxin-3	49	6850	1,67	6850
FGF-2	75	45000	11	45000
Fractalkine	59	9250	2,26	9250
G-CSF/CSF-3	42	52500	13	52500
GM-CSF	44	59700	15	59700
Gro-alpha/KC	61	16700	4,08	16700
HGF	46	21600	5,27	21600
IFN-alpha	48	29500	7,2	29500
IFN-gamma	43	54000	13	54000
IL-10	28	7350	1,79	7350
IL-12p70	34	26900	6,57	26900
IL-13	35	20000	4,88	20000
IL-15	65	12000	2,93	12000
IL-16	70	63300	15	63300
IL-17A	36	15800	3,86	15800
IL-18	66	39500	9,64	39500
IL-1alpha	62	7100	1,73	7100
IL-1beta	18	25600	6,25	25600
IL-2	19	30700	7,5	30700
IL-20	81	58900	14	14725
IL-21	72	38700	9,45	38700
IL-22	76	67700	17	67700
IL-23	63	54400	13	54400
IL-2R	9	397600	97	397600
IL-3	73	112400	27	112400
IL-31	37	78900	19	78900
IL-4	20	44600	11	44600
IL-5	21	29900	7,3	29900
IL-6	25	58100	14	58100
IL-7	26	3100	0,76	3100
IL-8	27	11700	2,86	11700
IL-9	52	32600	7,96	32600
IP-10	22	8200	2	8200
I-TAC	57	42900	10	42900
LIF	15	16400	4	16400
MCP-1	51	10700	2,61	10700
MCP-2	8	4150	1,01	1038
MCP-3	68	16200	16	16200
M-CSF	67	57700	14	57700
MDC/CCL22	87	56500	14	56500
MIF	53	3800	0,93	3800
MIG	69	30200	7,37	30200
MIP-1alpha	12	14500	3,54	3625
MIP-1beta	47	34300	8,37	8575
MIP-3alpha	56	39600	9,67	39600
MMP-1	64	17200	4,2	17200
SCF	39	18900	4,61	18900
SDF-1alpha	13	185400	45	185400
TNF-alpha	45	51600	13	51600
TNF-beta	54	23600	5,76	23600
TNF-RII	85	10700	2,61	10700
TRAIL	58	13700	3,34	13700
TSLP	80	19500	4,76	19500
Tweak	97	393900	96	393900
VEGF-A	78	27400	6,69	27400

Legend: Std – standard; LLOQ – lower limit of quantification; UPOQ – upper limit of quantification

ProcartaPlex™ Human Immuno-Oncology Checkpoint Panel 1, 14plex; Panel 2, 14plex; Panel 3, 10plex

Analyte	Bead Number	[Std1] (pg/mL)	LLOQ (pg/mL)	ULOQ (pg/mL)
Panel 1				
BTLA	52	492500	120	492500
CD137/4-1BB	26	47400	12	47400
CD152/CTLA4	33	34700	8,47	34700
CD27	27	23900	5,83	23900
CD28	15	132800	32	132800
CD80	61	150700	37	150700
GITR	57	85500	21	85500
HVEM	36	59700	15	59700
IDO	46	13200	3,22	13200
LAG-3	47	43700	11	43700
PD-1	65	30000	7,32	30000
PD-L1	66	14500	3,54	14500
PD-L2	67	189300	46	189300
TIM-3	14	303700	74	303700
Panel 2				
Arginase	51	50000	12	50000
E-Cadherin	44	148600	36	148600
MICA	18	53100	13	53100
MICB	21	30000	7,32	30000
Nectin-2 (CD112)	29	152800	37	152800
NT5E (CD73)	30	184800	45	184800
Perforin	53	258700	63	258700
PVR (CD155)	56	222500	54	222500
Siglec-7	12	63000	62	63000
Siglec-9	13	7600	1,86	7600
Tactile (CD96)	35	291000	71	291000
ULBP-1	73	584700	143	584700
ULBP-3	77	81000	79	81000
ULBP-4	78	337100	82	337100
Panel 3				
E-Cadherin	44	148600	36	148600
B7-H6	42	485400	119	485400
CD134 (OX40)	55	30800	7,52	30800
CD276 (B7-H3)	72	584700	571	584700
CD47 (IAP)	74	25100	6,13	25100
CD48 (BLAST-1)	19	116400	28	116400
Galectin-9	38	9100	2,22	9100
ICOS Ligand	34	37000	9,03	37000
S100A8/A9	76	249200	61	249200
TIMD-4	39	248600	61	248600
VISTA (B7-H5)	64	35400	8,64	35400
B7-H6	42	485400	119	485400

Supplementary file S6. Absolute frequency (AF, A) and relative frequency (RF, B) of major leucocyte (LEU) subpopulations, including granulocytes (GR), monocytes (MO, lymphocytes (LY), dendritic cells (DC), and myeloid-derived suppressor cells (MDSC).

A

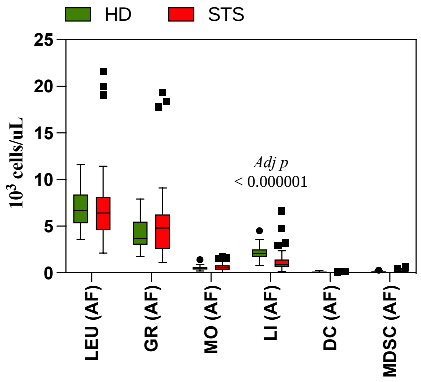

B

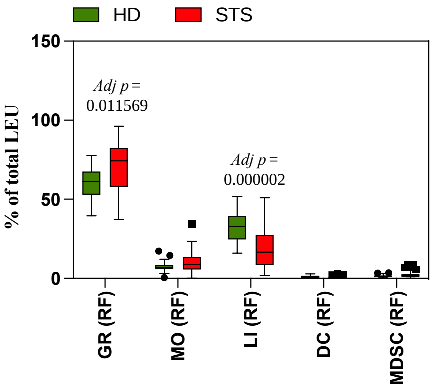

Supplementary file S7. Absolute frequency (AF, A) and relative frequency (RF, B) of major lymphocyte subpopulations, including T cells, NK cells, NKT-like cells, and B cells.

A

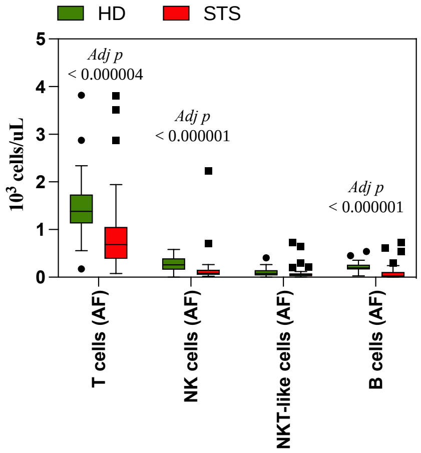

B

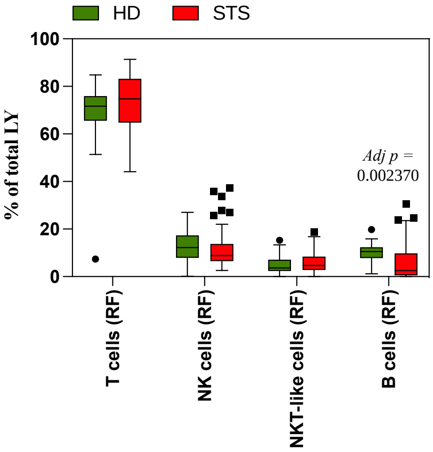

Supplementary file S8. Absolute frequency (AF, A) and relative frequency (RF, B) of T cells subpopulations, including double negative (DN), double positive (DP), CD4 and CD8 T cells.

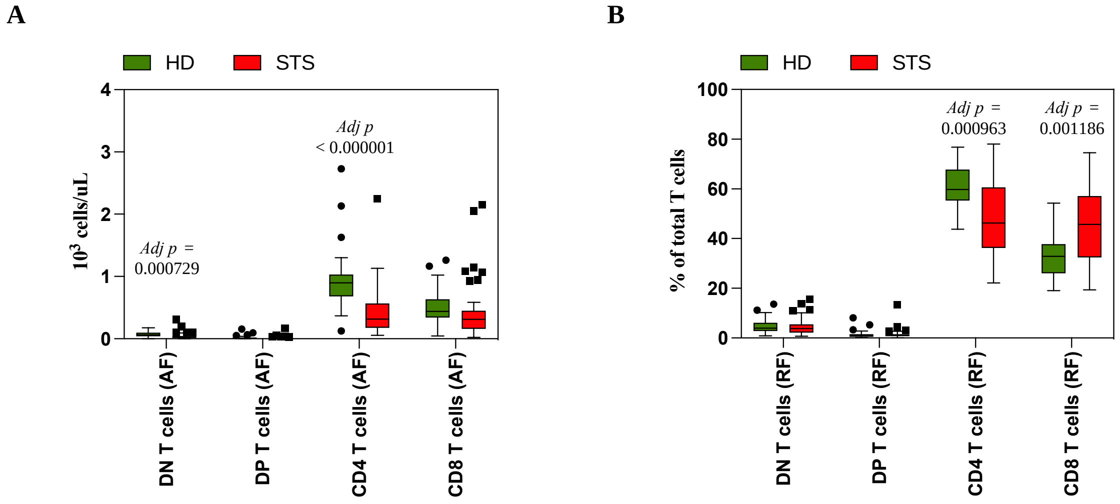

Supplementary file S9. Relative frequency of CD4 T cells subpopulations related to maturation (A), including naïve, central memory (CM), effector memory (EM), and effector memory expressing CD45RA (EMRA), and differentiation (B), including Th1, Th2, Th7, and regulatory T (Treg) cells. Relative frequency of CD8 T cells subpopulations related to maturation (C), including naïve, naïve low, CM, EM, EMRA low, and EMRA.

A

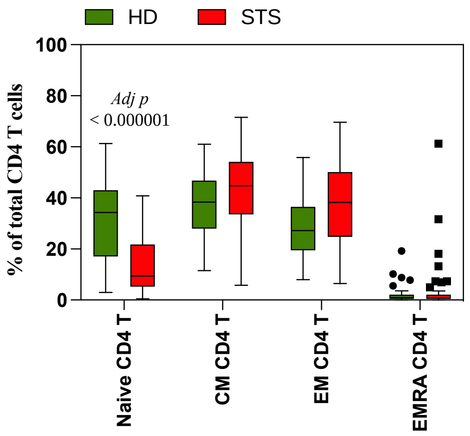

B

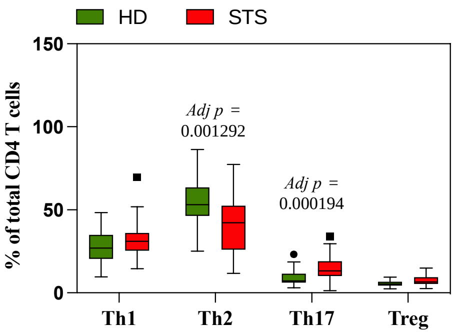

C

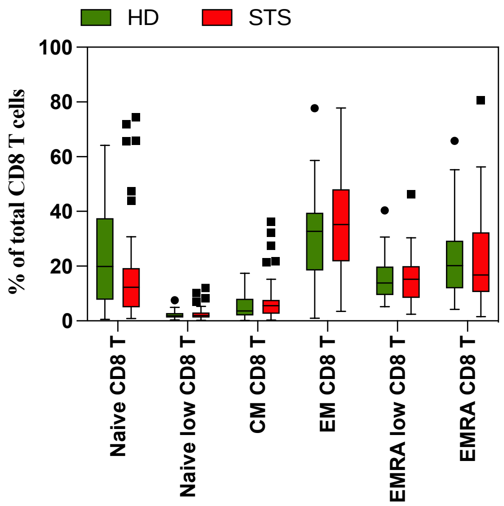

Supplementary file S10. Multivariate Cox analysis using stepwise method with forward selection (likelihood ratio) for the selected variables included in the clustering analysis adjusting for clinical data, including tumor site (extremity, trunk non-RPS, RPS, gynecologic, head & neck); tumor grade (low grade (non-metastatic primary); high grade (non-metastatic primary recurrent; metastatic primary; metastatic recurrent); age at diagnosis; and age at collection time.

**Multivariate Cox Analysis – Forward Stepwise Conditional (LR) Method**										**Univariate Cox Analysis**				
**Omnibus Tests of Model Coefficients**				**Variables in the Equation**										
Step	Overall (score)				B	Sig.	Exp(B)	95.0% CI)		B	Sig.	Exp(B)	95.0% CI	
	Chi-square	*df*	*Sig.*					*Lower*	*Upper*				*Lower*	*Upper*
1a	11.503	1	0.001	CD40LG	-1.439	0.015	0.237	0.075	0.755		0.037	0.312	0.105	0.930
1b	12.742	2	0.002	PMN-MDSC	0.746	0.088	2.108	0.894	4.968		0.001	1.050	1.020	1.082
				CD40LG	-2.429	0.040	0.088	0.009	0.897					

Supplementary file S11. Log-rank test and Kaplan Mayer curves for survival analysis of immunotypes considering the time after diagnosis (TAD) as the variable time.

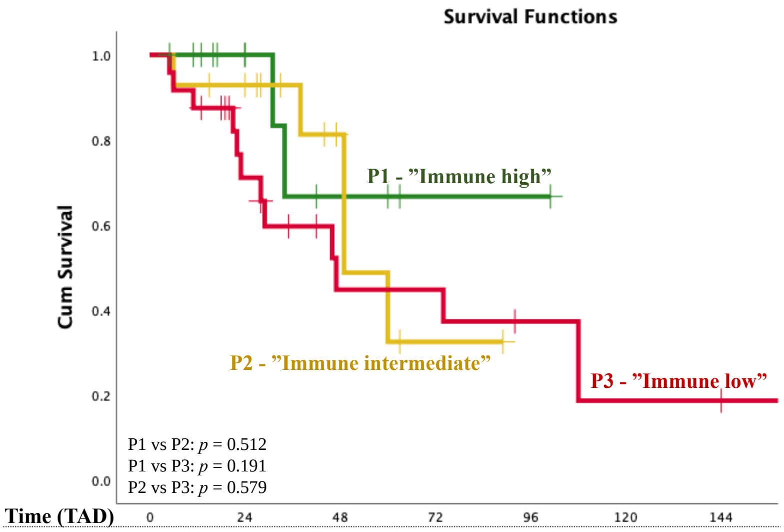

Number at risk, cumulative events (number censored)

**P1**	13 (0)	6 (7)	3 (8)	1 (10)	1 (10)	0 (11)	0 (11)
**P2**	14 (0)	11 (2)	5 (7)	1 (8)	0 (9)	0 (9)	0 (9)
**P3**	24 (0)	13 (5)	6 (8)	6 (8)	4 (9)	2 (9)	1 (10)
